# Selenite modulates phenotype-dependent epithelial–mesenchymal plasticity in pancreatic ductal adenocarcinoma: integrated in vitro analyses and patient-derived ex vivo tissue-slice cultures

**DOI:** 10.1186/s13046-026-03778-4

**Published:** 2026-07-13

**Authors:** Ozan Aricak, Wenyang Shi, Carlos Fernández Moro, Mehran Ghaderi, Joakim Dillner, Jonas Fuxe, Mikael Björnstedt, Tímea Szekerczés

**Affiliations:** 1 Division of Pathology, Department of Laboratory Medicine, Karolinska Institutet, Stockholm, Sweden; 2 Department of Clinical Pathology and Cancer Diagnostics, Karolinska University Hospital, Stockholm, Sweden; 3 Department of Clinical Science, Intervention and Technology (CLINTEC), Karolinska Institutet, Stockholm, Sweden

**Keywords:** Pancreatic ductal adenocarcinoma, Sodium selenite, Epithelial–mesenchymal transition, Ex vivo tissue-slice culture, Extracellular matrix remodeling

## Abstract

**Background:**

Pancreatic ductal adenocarcinoma (PDAC) is characterized by profound therapy resistance, desmoplasia, and phenotypic plasticity. While sodium selenite is a redox-active compound with reported tumor-selective cytotoxicity, its impact on epithelial–mesenchymal transition (EMT) states in PDAC remains insufficiently defined. We investigated whether selenite modulates EMT-associated phenotypes in a dose- and context-dependent manner using complementary in vitro and patient-derived ex vivo models and linked marker shifts to histological tumor regression in PDAC tissue slices.

**Methods:**

Three PDAC cell lines spanning distinct baseline EMT states (PANC-1, HPAF-II, Capan-2) were profiled by quantitative immunofluorescence for a predefined epithelial (EpCAM, cytokeratin, E-cadherin) and mesenchymal-associated (AHNAK2, ITGAV, vimentin) protein panel, with or without TGF-β pretreatment. Ex vivo tissue slices from treatment-naïve, non-metastatic PDAC resections (*n* = 10) were cultured in a within-patient paired design (0 h, 24 h, 48 h controls; 5 or 15 µM selenite during the second 24 h). Histological tumor regression was scored by a blinded pancreatic pathologist (Evans; CAP), compartment-resolved marker expression was quantified by multiplex immunofluorescence, and paired RNA sequencing was performed in a subset of donors (*n* = 4).

**Results:**

In vitro, responses were phenotype-contingent: the epithelial-biased HPAF-II and Capan-2 lines showed partial epithelial reinforcement and a consistent reduction of mesenchymal markers (particularly ITGAV, vimentin), whereas the mesenchymal-biased PANC-1 exhibited limited modulation. TGF-β pretreatment attenuated epithelial-promoting effects while suppression of mesenchymal markers was retained in a cell line–dependent manner. In ex vivo slices, selenite induced a dose-dependent improvement in histological tumor regression and increased the tumor-compartment epithelial–mesenchymal ratio at 15 µM, driven mainly by epithelial-marker upregulation, while mesenchymal markers showed no uniform suppression. At 15 µM, transcriptomics revealed a compact treatment response characterized by downregulation of basement-membrane/ECM modules without broad reversal across EMT gene sets; EMT scoring indicated heterogeneous shifts at 5 µM and a more consistent epithelial-leaning shift at 15 µM in most donors.

**Conclusions:**

Selenite was associated with reinforcement of epithelial features mainly in malignant PDAC compartments in a baseline-state-, dose-, and context-dependent manner, with higher-dose phenotypic shifts occurring alongside stronger histological tumor response in clinically proximal models.

**Trial registration:**

Not applicable.

**Supplementary Information:**

The online version contains supplementary material available at 10.1186/s13046-026-03778-4.

## Background

Pancreatic ductal adenocarcinoma (PDAC) remains one of the deadliest malignancies worldwide, with rapid local progression, early dissemination, and limited durable responses to systemic therapy [1]. Even with modern multimodal management, overall five-year survival is still ~ 10% [1–3]. Surgical resection is the only potentially curative option; however, fewer than 20% of patients have resectable disease at the time of diagnosis [1]. For most patients, combination chemotherapy regimens such as FOLFIRINOX or gemcitabine plus nab-paclitaxel provide only modest benefit and are limited by cumulative toxicity and resistance [2, 4]. Chemoresistance, a dense desmoplastic stroma, and marked intratumoral heterogeneity further compromise outcomes [1, 4]. These challenges highlight a need for approaches that exploit PDAC biology and can be tested in clinically proximal models that preserve key aspects of the human tumor context.

A prominent vulnerability in cancer is redox imbalance. High metabolic activity, mitochondrial dysfunction, and altered antioxidant defenses impose chronic oxidative stress, which can create sensitivity to additional redox disruption [5, 6]. Sodium selenite is an inorganic redox-active compound with reported tumor-selective cytotoxicity linked to reactive oxygen species generation and downstream activation of cell-death pathways, including apoptosis and ferroptosis [7, 8]. Preclinical studies have shown antitumor activity across multiple solid tumors [7–9], including PDAC, and selenite has induced tumor-cell death in human ex vivo tissue cultures [10]. Early-phase clinical studies of intravenous selenite have primarily established pharmacokinetics and tolerability, supporting the feasibility of high-dose administration in patients [11, 12].

While selenite’s cytotoxic redox-disrupting effects are increasingly well characterized [7], its impact on tumor plasticity, particularly epithelial–mesenchymal transition (EMT), is less well understood. Redox signaling intersects with core EMT pathways, including transcriptional control and cell-adhesion programs, suggesting that selenite-induced reactive oxygen species (ROS) changes could influence EMT dynamics in parallel with cytotoxic effects [13]. ROS-dependent signaling has also been linked to several EMT-regulatory pathways, including TGF-β, NF-κB, and integrin/FAK-associated signaling [13, 14], supporting a potential overlap between redox disruption, EMT-associated phenotypic modulation, and tumor-cell injury. In addition, the xCT/SLC7A11 axis, which contributes to cellular redox buffering and selenite sensitivity, has recently been linked to EMT-associated phenotypes in cancer, including PDAC, providing further biological context for a possible connection between redox vulnerability and EMT plasticity [15, 16].

EMT is not a binary switch but a continuum in which epithelial tumor cells progressively lose polarity and cell–cell junctions and acquire mesenchymal traits that support motility, invasion, and therapy resistance [17, 18]. In PDAC, EMT-associated programs correlate with aggressive behavior, early dissemination, and poor outcomes, and intermediate states along the continuum are common [18, 19]. Reversal toward epithelial features (mesenchymal–epithelial transition, MET) has been proposed as a route to reduce invasive behavior and improve treatment responsiveness, although the extent and clinical meaning of such shifts are context-dependent [20, 21]. A key translational challenge is therefore to determine whether redox-active agents can modulate EMT-associated phenotypes in PDAC and whether such effects depend on baseline state and microenvironmental context.

To capture phenotype-level modulation across model systems, we quantified a predefined six-marker panel covering epithelial identity and cell–cell adhesion (EpCAM, Cytokeratin (CK), E-cadherin (E-cad)) together with mesenchymal and invasion-associated features represented by AHNAK2, Integrin Subunit Alpha V (ITGAV), and Vimentin, which are linked to polarity, adhesion, migration, and invasion [17, 21–23]. This multi-marker approach was designed to detect coordinated shifts along the EMT axis rather than relying on a single surrogate marker, consistent with current recommendations that EMT status should be assessed using multiple molecular readouts [24]. Each component has been implicated in PDAC progression and therapy resistance [22, 23, 25–29]. Because transforming growth factor–β (TGF-β) is a major EMT driver in PDAC and intersects with oxidative imbalance in the tumor microenvironment, it also provides a clinically relevant EMT-inducing context for testing redox-based modulation [18]. Whether redox-based agents such as selenite can modulate TGF-β–induced EMT, or suppress mesenchymal features independently, remains unclear.

Here, we investigated whether selenite modulates EMT-associated phenotypes in PDAC in a dose- and context-dependent manner, including under EMT-inducing conditions. We integrated in vitro profiling of three PDAC cell lines with distinct baseline EMT states, with and without TGF-β pretreatment, together with patient-derived ex vivo PDAC tissue-slice cultures. The ex vivo model preserves tissue architecture and stromal context, enabling compartment-resolved protein readouts with parallel assessment of histological tumor regression, and paired transcriptomic profiling in a subset of donors. Together, this design tests selenite not only as a cytotoxic redox disruptor but also as a modulator of PDAC phenotypic plasticity in a clinically relevant tissue context.

## Methods

### Study design

This study assessed whether sodium selenite modulates epithelial–mesenchymal features in PDAC in a dose- and context-dependent manner using complementary in vitro and ex vivo models. The primary outcomes were changes in a predefined EMT-related protein panel (EpCAM, CK, E-cad, AHNAK2, ITGAV, Vimentin) and the epithelial–mesenchymal (EM) ratio, with histological tumor regression in ex vivo slices (Evans grade and the College of American Pathologists (CAP) criteria) and paired transcriptomic responses (RNA-seq–based differential expression and EMT scoring) as additional outcomes.

In vitro, three PDAC cell lines (PANC-1, HPAF-II, Capan-2) were assessed under basal conditions and after TGF-β pretreatment, with each condition repeated in ≥ 3 independent experiments using replicate wells. Ex vivo analyses were performed on chemotherapy-naïve, non-metastatic PDAC resections (*n* = 10) and processed in a within-patient paired slice-culture design including baseline (0 h), culture controls (24 h and 48 h), and selenite exposure (5 or 15 µM during the second 24 h), enabling matched comparisons within each donor. RNA-seq was performed in four patients based on material availability and predefined quality requirements. Targeted molecular analysis was additionally performed on available formalin-fixed, paraffin-embedded (FFPE) tumor tissue for descriptive molecular characterization of the ex vivo cohort.

Samples were included if review of hematoxylin and eosin (H&E)-stained sections confirmed adequate tissue integrity and sufficient PDAC content for evaluation. Regions with sparse representation (ductal epithelium and PanIN) were not included in paired statistical analyses; analyses focused on tumor and acinar compartments. Sample size was determined by tissue availability and quality criteria. Histological tumor regression scoring was conducted by a pancreatic specialist pathologist blinded to condition and timepoint.

### Cell culture and cytotoxicity assays

Three human PDAC cell lines were used: PANC-1 (CRL-1469) and HPAF-II (CRL-1997) were obtained from the American Type Culture Collection (ATCC), while Capan-2 (ACC 245) cells were from the Leibniz Institute DSMZ-German Collection. All cell lines were cultured in EMEM (30-2003, ATCC) supplemented with 10% fetal bovine serum (FBS; #10500-064, Thermo Fisher Scientific) without antibiotics, at 37 °C with 5% CO₂. For EMT induction, cells were pretreated with TGF-β (#7754-BH, R&D Systems) at 10 ng/mL for 48 h.

To determine selenite IC₅₀/IC₂₅ values, untreated and TGF-β-pretreated cells were seeded into 96-well plates (200/300/400 cells/mm² for PANC-1/HPAF-II/Capan-2). Sodium selenite (#214485; Sigma-Aldrich) was added 24 h after seeding as a 9-point dilution series (maximum 100 µM) and incubated for 24 h; in TGF-β–pretreated arms, TGF-β remained present during selenite treatment. Viability was measured using CellTiter-Glo^®^ (#G7571; Promega) on a CLARIOstar FLx100 luminometer (BMG Labtech) and IC₂₅/IC₅₀ values were derived by nonlinear regression. Each condition was assessed in ≥ 3 independent experiments (biological replicates), each including replicate wells (technical replicates).

### Immunofluorescence staining of EMT markers

Cells were seeded into 96-well plates (#89606; Ibidi) and treated for 24 h with selenite at IC₂₅ and IC₅₀ concentrations, with or without TGF-β pretreatment (10 ng/mL, 48 h; maintained during selenite exposure). Cells were fixed in 4% paraformaldehyde, permeabilized with 0.2% Triton X-100, and blocked with 5% normal donkey serum, followed by overnight incubation at 4 °C with primary antibodies against epithelial (EpCAM, CK, E-cad) and mesenchymal (AHNAK2, ITGAV, Vimentin) markers (Table 1). Cross-adsorbed Alexa Fluor secondary antibodies (488 anti-mouse, #A32766; 647 anti-rabbit, #A32795) and 568 phalloidin (#A12380; Thermo Fisher Scientific) were applied for 1 h, followed by nuclear counterstaining with Hoechst 33342.

**Table 1:** Primary antibodies used for immunofluorescence experiments. Primary antibodies used in vitro immunofluorescence staining and ex vivo multiplex immunofluorescence experiments. (Dilutions indicate the working concentrations used in the experiments described in Methods.)

Marker	Antibody (clone/type)	Host	Manufacturer	Catalog no.	Dilution
EpCAM	EpCAM (VU1D9)	Mouse	Cell Signaling	2929 S	1:100
E-cad	Anti-CDH1 Antibody (CL1170)	Mouse	Atlas Antibodies	AMAb90862	1:200
CK	Anti-pan Cytokeratin [AE1/AE3]	Mouse	Abcam	ab80826	1:50
AHNAK2	Anti-AHNAK2 polyclonal	Rabbit	Atlas Antibodies	HPA002940	1:200
Vimentin	Anti-Vimentin polyclonal	Rabbit	Merck	HPA001762	1:200
ITGAV	Anti-Integrin alpha V antibody [EPR19669]	Rabbit	Abcam	ab208012	1:200

Images were acquired using a Nikon A1R confocal microscope with Perfect Focus System enabled to maintain focal stability during acquisition. Samples within the same staining combinations were captured under identical exposure settings and processed in the same manner. Cells were segmented on the nuclear channel and mean per-cell fluorescence intensities were extracted per marker; background-corrected values were aggregated per well. Statistical analyses and plots used per-well means. The EM ratio was calculated from well-level marker means as the geometric mean of epithelial markers (EpCAM, CK, E-cad) divided by the geometric mean of mesenchymal markers (AHNAK2, vimentin, ITGAV), with higher values indicating a more epithelial state. Per-well EM ratios were summarized across replicate wells and independent experiments.

### Patient cohort and sample selection criteria

Treatment-naïve PDAC tissue was collected from non-metastatic patients (*n* = 10) undergoing upfront surgical resection at Karolinska University Hospital (Stockholm, Sweden) between May 2022 and March 2024. Clinicopathological data were extracted from final histopathology reports, and TNM stage and margin status were assigned according to American Joint Committee on Cancer (AJCC; 8^th^ edition). The study was conducted under ethical approval from the Swedish Ethical Review Authority (Etikprövningsmyndigheten) and in accordance with the Declaration of Helsinki; all patients provided written informed consent. Samples were pseudonymized prior to researcher access.

### Targeted molecular analysis

Mutational analysis using the Oncomine™ Focus DNA Assay was performed at the Clinical Molecular Cancer Diagnostics Unit in Huddinge, Department of Clinical Pathology and Cancer Diagnostics, Karolinska University Hospital. Targeted molecular analysis was performed on FFPE tumor tissue from all 10 cases in the ex vivo cohort. Tumor-containing areas were reviewed by a pancreatic specialist pathologist on corresponding H&E-stained sections before DNA extraction, and all analyzed samples contained > 15% tumor tissue. Genomic DNA was extracted from one 10-µm FFPE tissue section using the Maxwell^®^ RSC FFPE DNA PLUS Kit (#AS1720; Promega, Madison, WI, USA) according to the manufacturer’s instructions. DNA concentration was quantified using a Qubit™ fluorometer (Thermo Fisher Scientific, Waltham, MA, USA). Fifty nanograms of DNA was used for library preparation and sequencing with the Oncomine™ Focus Assay and Chef Ready Reagents on the Ion GeneStudio™ S5 System (Thermo Fisher Scientific). The assay interrogates hotspot mutations in 35 cancer-related genes. Data processing, alignment, and variant calling were performed using Torrent Suite™ Software (Thermo Fisher Scientific) with the standard automated analysis workflow.

### Ex vivo tissue-slice culture, selenite treatment, and histological evaluation

Patient-derived PDAC tissue-slice cultures were established as previously described [30]. Briefly, tumor regions were selected by a pancreatic specialist pathologist and sectioned into 350 μm slices using a vibrating-blade microtome (VT1200S, Leica). Slices were maintained on 0.4 μm Millicell membrane inserts (30 mm, Merck) in culture dishes using CMRL medium (glutamine-free, Thermo Fisher Scientific) supplemented as described [30]. The first slice was fixed immediately as the 0 h baseline. Additional slices were fixed after 24 h as culture controls and after 48 h as untreated controls; treated slices received 5 or 15 µM sodium selenite during the second 24 h (24–48 h), in duplicate per dose, with untreated slices processed in parallel.

H&E-stained sections were reviewed to confirm culture adequacy and sufficient PDAC content. A pancreatic specialist pathologist, blinded to condition and timepoint, scored histological tumor regression per slice using Evans grade [31] and CAP criteria [32] adapted to ex vivo tissue slices. Tumor cell death was defined by cytoplasmic degeneration/necrosis and nuclear pyknosis/karyolysis [33, 34]. For downstream analyses, regression was dichotomized as low (Evans I–IIa or CAP 3) versus high (Evans IIb–IV or CAP 0–2) [33].

### Multiplex immunofluorescence staining and image analysis

FFPE sections (3–4 μm) were cut from 0 h baseline, 24 h culture control, 48 h untreated control, and 48 h selenite-treated (5 or 15 µM) tissue slices, and mounted on Ultra SuperFrost Plus™ slides (Thermo Fisher Scientific). Multiplex immunofluorescence staining was performed at Karolinska Institutet FENO Core Facility using the Opal™ 7-Color Automation IHC Kit (#NEL821001KT; Akoya Biosciences) on a Leica BOND RXm (Leica Biosystems) following standard deparaffinization and heat-induced epitope retrieval. Primary antibodies targeted epithelial (EpCAM, CK, E-cad) and mesenchymal markers (AHNAK2, Vimentin, ITGAV) (Table 1), each assigned to a distinct Opal fluorophore. Slides were counterstained with DAPI and mounted in antifade medium. Whole-slide imaging was performed on a Vectra 3 System (Akoya Biosciences), regions of interest were selected at 4× and acquired multispectrally at 10×, followed by spectral unmixing in inForm v2.6.0.

Quantitative image analysis was performed in QuPath v0.5.1. A pancreatic specialist pathologist annotated tumor, PanIN, acinar parenchyma, ductal epithelium, and mesenchymal elements (vessels, nerves) guided by matched H&E slides. Compartment boundaries were defined based on nuclear morphology, glandular architecture, and stromal context, enabling marker quantification to be performed separately within each annotated region. Nuclei were segmented on the DAPI channel (preferred pixel size: 1 μm) and mean per-cell fluorescence intensity was extracted for each marker within each annotated region independently.

To correct technical variation, intensities were standardized per patient and per marker: for each patient, the marker-wise mean and standard deviation (SD) were computed across that patient’s five tissue samples and all annotation types; raw values were then transformed into Z-scores. Within each sample, Z-scores were averaged by marker and annotation type, yielding one normalized value per tissue compartment per patient. This within-patient standardization supports paired comparisons across conditions while dampening baseline and region-size effects. EMT status was summarized as EM ratio. Because Z-scores can be negative, a constant c was added (Z′ = Z + c, with c chosen so that all Z′ > 0) before computing geometric means.

### RNA sequencing and bioinformatic analysis

RNA-seq was performed from four patient-derived tissue cultures (OT127, OT138, OT142, OT146). For each patient, four conditions were profiled: 0 h baseline, 48 h treatment control, and 48 h cultures treated with selenite during the second 24 h (5 or 15 µM). Total RNA was extracted from 10-µm FFPE sections (Maxwell RSC FFPE RNA Kit, #AS1440, Promega) and quantified on a Qubit 4.0 fluorometer using the RNA HS Assay Kit (#Q32852, Thermo Fisher Scientific). Libraries were prepared from up to 50 ng RNA using SMARTer Total RNA-Seq Kit v2.5 Pico Input Mammalian (Takara Bio) [35] and sequenced on an Illumina NextSeq 500/550 (Mid Output Kit v2.5, #20024904, 2 × 75 bp), yielding a median of ~ 27 million total reads (~ 13.5 million read pairs) per library.

FASTQ processing was performed in Chipster (CSC, Finland) [36]. Reads were quality-checked (FastQC/MultiQC), trimmed (Trimmomatic v0.39) [37], aligned to GRCh38 (STAR v2.7.10a) [38] and quantified at gene level (HTSeq-count v0.13.5; GRCh38.109; stranded “reverse”). Differential expression was assessed in edgeR using a paired design (patient + group), TMM normalization, and Benjamini–Hochberg FDR control (FDR < 0.05). Two contrasts were tested: 48 h control vs. 0 h (culture-time effects) and 5 or 15 µM vs. the matched 48 h control (treatment effects). EMT-focused analyses were performed separately: EMT scoring used the pre-DE expression matrix restricted to a curated EMT reference list (Additional file 1: Table S1), whereas functional enrichment was performed on the resulting DE gene lists.

#### EMT gene set curation

An EMT-focused reference list was assembled by merging EMTome-curated gene sets [39] including Hallmark EMT [40], PDAC core EMT panel [41], pan-cancer EMT signature [42] and by adding genes corresponding to the multiplex protein panel (EPCAM, CDH1, VIM, ITGAV, AHNAK2, and panCK-associated KRTs). After HUGO Gene Nomenclature Committee (HGNC) harmonization and de-duplication, the final list comprised 448 unique genes annotated by source sets (Additional file 1: Table S1).

#### EM score frameworks

EMT scoring used the pre-differential expression (DE) raw-count matrix converted to log₂CPM; gene-wise z-scores were computed across the patient-matched 48 h control, 5 µM, and 15 µM profiles and used as input for all frameworks. A multinomial logistic regression (MLR) style proxy was calculated from log₂(VIM/CDH1) and − log₂(CLDN7), each z-standardized across matched profiles, averaged with equal weights, and linearly mapped to the conventional 0–2 MLR range (higher = more mesenchymal-like) [43]. The PDAC core 22-gene score was computed as EM = E−M, where E and M represent the mean z-score across a priori epithelial- and mesenchymal-labeled genes, respectively (higher = more epithelial-like) [39, 41]. A genome-wide extension was generated from the curated 448-gene list by propagating E/M labels using control-only training: in the four patient-matched 48 h controls, reference E and M vectors were defined by the 22-gene panel, and each candidate gene was assigned to the class with the stronger positive Pearson correlation to these vectors; labels were then fixed and applied to all samples to compute per-sample E and M means and EM = E−M (higher = more epithelial-like).

#### Functional enrichment analysis

Significantly up- and down-regulated gene lists (per contrast) were analyzed in DAVID Bioinformatics Resources (v2024) [44, 45]. Enrichment was queried across GO_BP/GO_CC/GO_MF (Direct) and pathway databases (KEGG, Reactome) using Functional Annotation Clustering with custom stringency (EASE = 0.05; kappa/term-overlap = 0.55; initial/final group membership = 3; multiple linkage = 0.5) and a Homo sapiens background. P-values were Benjamini–Hochberg adjusted; only terms with BH FDR (q) ≤ 0.05 were interpreted. Where overlapping terms were returned, related annotations were summarized into clusters, and a representative term (reported with Fold Enrichment, gene count, and q-value) was retained for the main text; full outputs are provided in Additional file 1: Tables S2–S3. When indicated, enrichment was repeated on EMT-filtered DE subsets derived from the curated EMT panel.

### Statistical analysis

All statistical analyses and data visualizations were performed in GraphPad Prism v10. Normality was assessed using Shapiro–Wilk and Kolmogorov–Smirnov tests to guide the choice of parametric versus non-parametric methods.

For in vitro experiments, dose–response curves were fitted using nonlinear regression to calculate IC_25_, IC_50_ values. For > 2 groups, one-way ANOVA with Bonferroni’s post hoc test (parametric) or Kruskal–Wallis with Dunn’s multiple comparisons test (non-parametric) was applied. For the ex vivo dataset, matched comparisons across treatment conditions (untreated vs. 5/15 µM selenite) and/or timepoints (0 h vs. 24 h vs. 48 h) were analyzed using a mixed-effects model. When required, Geisser–Greenhouse correction was applied, and Tukey’s post hoc test was used for multiple comparisons.

Clinicopathological associations were evaluated by summarizing treatment effects as delta values (treated - patient-matched untreated control) and comparing deltas between clinical subgroups using two-sided t-tests or Wilcoxon tests, as appropriate. Evans and CAP tumor-regression scores were analyzed analogously. Correlations with clinicopathological variables used Pearson’s correlation for normally distributed continuous variables and Spearman’s rank correlation for non-normal or ordinal variables. A p-value ≤ 0.05 was considered statistically significant.

## Results

### In vitro EMT phenotypes and selenite responses in PDAC

#### Baseline EMT profile and selenite sensitivity

**Fig. 1:**
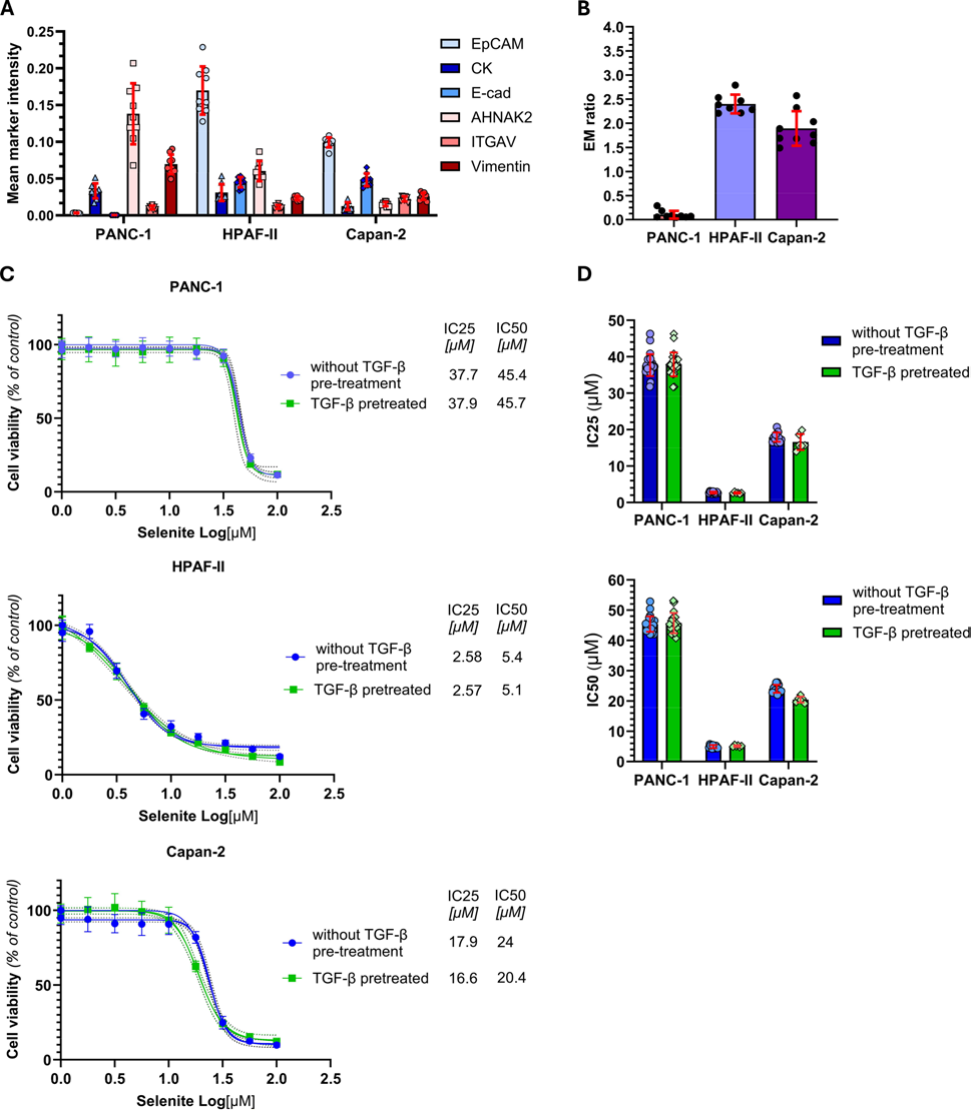
Baseline EMT profiles and selenite sensitivity in PDAC cell lines.** A** Baseline immunofluorescence quantification of epithelial markers (EpCAM, CK, E-cad) and mesenchymal markers (AHNAK2, ITGAV, Vimentin) in PANC-1, HPAF-II, and Capan-2 cells. Points represent independent experiments; bars show mean ± SD. **B** Epithelial-to-mesenchymal (EM) ratio per experiment, calculated as the geometric mean of epithelial marker intensities divided by the geometric mean of mesenchymal marker intensities (higher values indicate a more epithelial phenotype). Points represent independent experiments; bars show mean ± SD. **C** Twenty-four–hour sodium selenite dose–response curves (cell viability) in untreated cells and after TGF-β pretreatment (10 ng/mL, 48 h). Points show mean ± SD of replicate wells; curves show nonlinear regression fits. IC_25_ and IC_50_ values derived from fitted curves are indicated for each condition. **D **Summary of IC_25_ (top) and IC_50_ (bottom) values across independent experiments in untreated and TGF-β–pretreated cells. Points represent independent experiments; bars show mean ± SD. Statistical comparisons used two-tailed unpaired t tests; no significant differences were detected (*p* > 0.05). Each condition was repeated in ≥ 3 independent experiments

Baseline immunofluorescence (IF) revealed distinct EMT states across the three lines (Fig. 1A). PANC-1 displayed a mesenchymal-leaning pattern with high Vimentin and AHNAK2 and low epithelial markers. HPAF-II and Capan-2 exhibited predominantly epithelial marker profiles, with higher EpCAM/E-cad and generally low Vimentin and ITGAV (with elevated AHNAK2 in HPAF-II) (Fig. 1A). Accordingly, the EM ratio was high in HPAF-II and Capan-2 and markedly lower in PANC-1 (Fig. 1B).

Dose–response curves indicated differential selenite sensitivity (Fig. 1C): HPAF-II was most sensitive, Capan-2 intermediate, and PANC-1 least sensitive. IC₅₀/IC₂₅ values from nonlinear fits (Fig. 1D) were used to define doses for subsequent assays. TGF-β pretreatment did not alter selenite sensitivity, as dose–response curves and IC₅₀/IC₂₅ estimates were comparable between pretreated and untreated conditions (Fig. 1C–D).

#### Selenite modulates EMT markers across cell lines

**Fig. 2:**
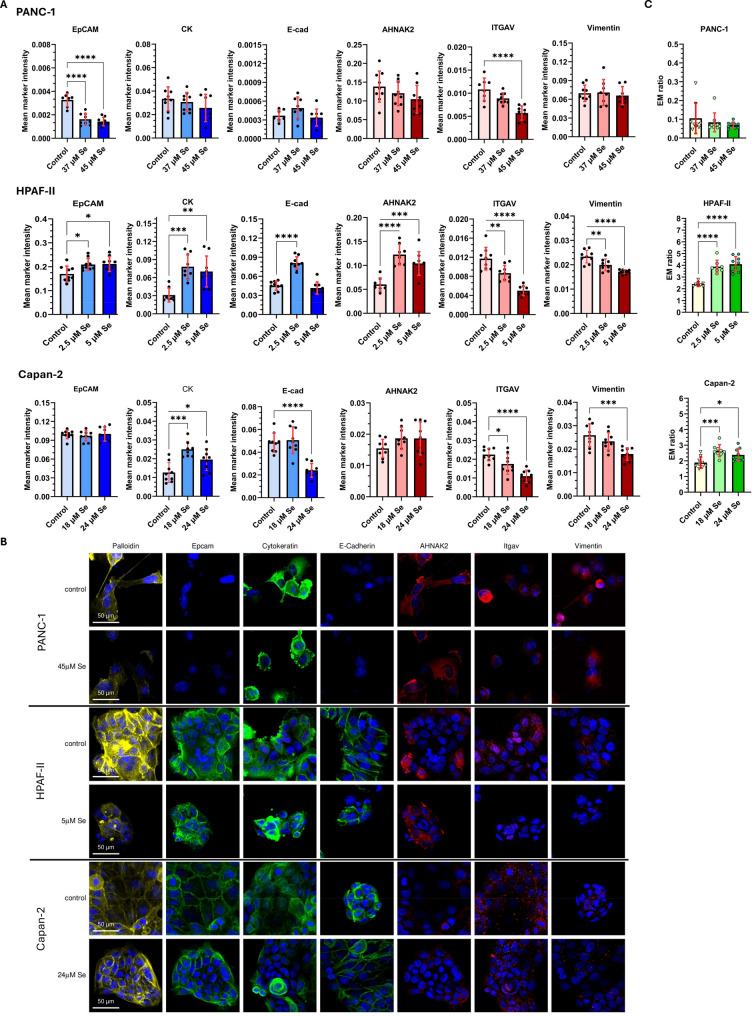
Selenite (Se) modulates EMT marker expression and EM ratio in PDAC cell lines. **A** Quantification of immunofluorescence marker intensities in PANC-1, HPAF-II, and Capan-2 after 24 h selenite treatment at two concentrations (PANC-1: 37 and 45 µM; HPAF-II: 2.5 and 5 µM; Capan-2: 18 and 24 µM; doses selected based on IC_25_/IC_50_ values in Fig. 1). Epithelial markers: EpCAM, CK, E-cad. Mesenchymal markers: AHNAK2, ITGAV, Vimentin. Bars show mean ± SD. **B** Representative immunofluorescence images of untreated controls and the higher selenite dose for each line (PANC-1: 45 µM; HPAF-II: 5 µM; Capan-2: 24 µM). Single-marker channels are shown as indicated; nuclei were counterstained with Hoechst and F-actin was visualized with phalloidin. Scale bars, 50 µm. **C** Epithelial–mesenchymal (EM) ratio per condition, calculated as the geometric mean of epithelial marker intensities divided by the geometric mean of mesenchymal marker intensities (higher values indicate a more epithelial phenotype). Bars show mean ± SD. Statistical comparisons in (**A**) and (**C**) were performed within each cell line using one-way ANOVA with Bonferroni’s multiple-comparisons test, comparing treated groups to the corresponding untreated control. *p < 0.05, **p < 0.01, ***p < 0.001, ****p < 0.0001. Data represent ≥ 3 independent experiments

Cells were exposed for 24 h to selenite at IC₂₅/IC₅₀ doses (PANC-1: 37/45 µM; HPAF-II: 2.5/5 µM; Capan-2: 18/24 µM). Marker responses were cell line–dependent (Fig. 2A) with representative high-dose images shown in Fig. 2B. In PANC-1, selenite reduced EpCAM in a concentration-dependent manner, while CK and E-cad were unchanged. Mesenchymal markers (AHNAK2, Vimentin) remained largely stable, with ITGAV showing a decrease only at 45 µM. In HPAF-II, EpCAM and CK increased at both doses; E-cad rose modestly at 2.5 µM but returned to baseline at 5 µM. Vimentin and ITGAV decreased, whereas AHNAK2 increased at both concentrations. In Capan-2, CK increased at both doses, while E-cad decreased slightly at 24 µM. ITGAV declined in a dose-dependent manner, Vimentin decreased at 24 µM, and AHNAK2 remained largely unchanged.

Consistent with these patterns, EM ratios increased moderately in HPAF-II and Capan-2 but remained low in PANC-1 (Fig. 2C), indicating a partial epithelial shift in the more epithelial-biased lines.

#### TGF-β modifies selenite effects on EMT

**Fig. 3:**
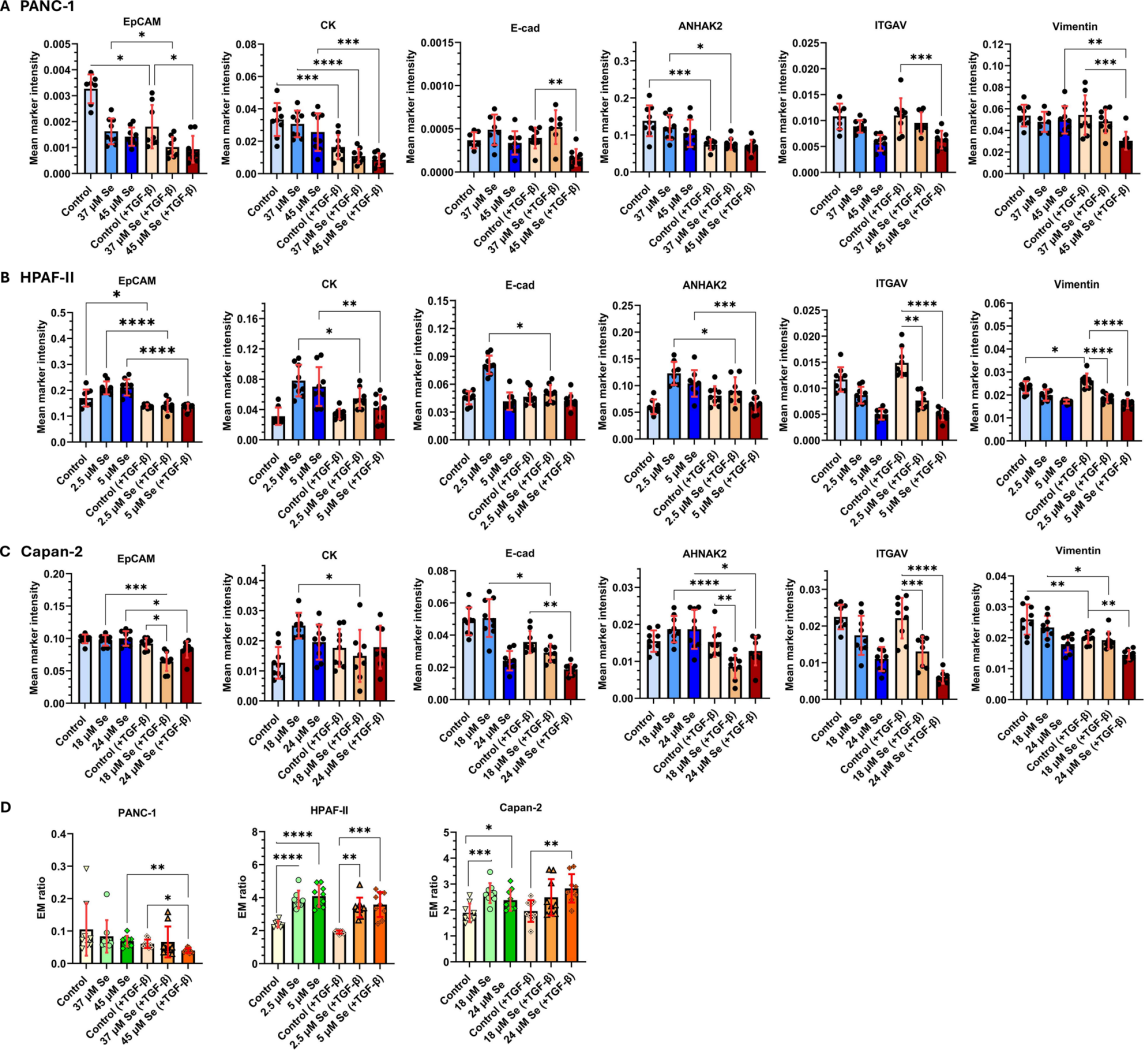
TGF-β priming reshapes selenite-induced EMT marker responses in PDAC cells. **A–C** Immunofluorescence quantification of epithelial (EpCAM, CK, E-cad) and mesenchymal (AHNAK2, ITGAV, Vimentin) markers in PANC-1 (**A**), HPAF-II (**B**), and Capan-2 (**C**). Cells were pretreated with TGF-β (10 ng/mL, 48 h) or left untreated, then exposed to sodium selenite for 24 h at doses approximating IC_25_/IC_50_ for each line (PANC-1: 37/45 µM; HPAF-II: 2.5/5 µM; Capan-2: 18/24 µM). In TGF-β–pretreated conditions, TGF-β was maintained during selenite exposure. Bars show mean ± SD, points represent independent experiments (≥3). **D** EM ratio for the same conditions, calculated as the geometric mean of normalized epithelial marker intensities divided by the geometric mean of mesenchymal marker intensities. Statistical comparisons were performed within each cell line using one-way ANOVA with Bonferroni’s post hoc test or Kruskal–Wallis with Dunn’s post hoc test, depending on data distribution. *p < 0.05, **p < 0.01, ***p < 0.001, ****p < 0.0001

In PANC-1, TGF-β pretreatment alone reduced EpCAM, CK and AHNAK2 versus matched controls. In pretreated cells, high-dose selenite further decreased EpCAM, E-cad, ITGAV and Vimentin. Under basal conditions, selenite also reduced EpCAM and ITGAV. For CK and AHNAK2, responses to the lower dose were attenuated by pretreatment, resulting in lower levels than the corresponding non-pretreated selenite condition; for CK, this attenuation was also evident at 45 µM (Fig. 3A).

In HPAF-II, TGF-β pretreatment decreased EpCAM and increased Vimentin (with a small, non-significant increase in ITGAV), consistent with a mesenchymal shift. Adding selenite after pretreatment reduced both Vimentin and ITGAV versus the TGF-β–pretreated control, countering the mesenchymal shift. By contrast, epithelial-promoting responses observed under basal selenite were blunted by pretreatment: in untreated cells, selenite increased EpCAM, modestly increased CK, upregulated E-cad at 2.5 µM, and elevated AHNAK2, whereas these effects were reduced or absent after TGF-β pretreatment (Fig. 3B).

In Capan-2, TGF-β pretreatment alone had a limited effect, with a slight decrease in Vimentin. Under basal conditions, selenite increased CK, reduced E-cad at 24 µM, decreased ITGAV, and lowered Vimentin at 24 µM, while EpCAM and AHNAK2 were largely unchanged. Under TGF-β pretreatment, the lower selenite dose decreased EpCAM and AHNAK2 and no longer increased CK; effects on E-cad, ITGAV and Vimentin were broadly similar to basal conditions (Fig. 3C).

Overall, selenite most consistently reduced the mesenchymal markers ITGAV and Vimentin, whereas epithelial-marker restoration was more variable and was generally attenuated by TGF-β pretreatment, particularly in PANC-1 and Capan-2. The EM ratio recapitulated these patterns (Fig. 3D). In PANC-1, selenite did not alter the EM ratio, whereas 45 µM after TGF-β pretreatment significantly reduced it versus both the pretreated control and 45 µM without pretreatment. In HPAF-II, pretreatment caused a mild, non-significant downward shift overall, yet selenite still increased the EM ratio at 2.5 and 5 µM. In Capan-2, 18 and 24 µM increased the EM ratio under basal conditions; after pretreatment, only 24 µM produced a significant increase relative to the pretreated control, while 18 µM showed a non-significant rise, suggesting reduced efficacy of the lower dose in the TGF-β–primed state.

### Ex vivo selenite responses in patient-derived PDAC tissue slices

#### Histological tumor regression and clinical correlates

Clinicopathological and available molecular characteristics of the cohort are summarized in Table 2. The cohort comprised 10 resected PDAC patients (5 women/5 men; median age 73.5 years, range 45–82; median tumor size 39 mm, range 25–50). Tumors were pT2–pT3; 9/10 were node-positive and all were M0 (AJCC 8^th^ edition: IB–III). MMR IHC was available in seven cases and showed proficient MMR in all assessed tumors. p53 IHC was available in six cases, showing overexpression in two cases, no overexpression in three cases, and an equivocal pattern in one case. Targeted Oncomine sequencing was available for all 10 cases and detected pathogenic KRAS mutations in all tumors, including codon 12 mutations in nine cases: p.Gly12Arg/G12R (*n* = 4), p.Gly12Val/G12V (*n* = 4), and p.Gly12Asp/G12D (*n* = 1), and one codon 13 mutation: p.Gly13Asp/G13D (*n* = 1).

**Table 2:** Clinicopathological and available molecular characteristics of the PDAC cohort used for ex vivo slice cultures (*n* = 10). Clinicopathological and available molecular variables are shown for treatment-naïve, non-metastatic patients included in the ex vivo slice-culture analyses

Parameter	Category	*n*	Details
Patient age *(years)*	–	–	45–82 (median: 73.5)
Patient sex	Male / Female	5 / 5	
Tumor size *(mm)*	–	–	25–50 (median: 39)
pT-stage	pT1 / pT2 / pT3 / pT4	0 / 5 / 5 / 0	
pN-stage	pN0 / pN1 / pN2	1 / 4 / 5	
TNM stage*	IB / IIB / III	1 / 4 / 5	
Lymph vessel invasion	L1 / L0	10 / 0	
Blood vessel invasion	V1 / V0	6 / 4	
Perineural invasion	Pn1 / Pn0	9 / 1	
Microscopic margin status	R1 / R0	10 / 0	(R1 defined as tumor at margin or < 1 mm)
Grade of differentiation	G1 / G2 / G3	0 / 6 / 4	(G1: well, G2: moderate, G3: poor)
Histological subtype **	Pancreatobiliary / Undifferentiated	8 / 2	(1 anaplastic, 1 sarcomatoid)
MMR status *(IHC)*	pMMR / Not assessed	7 / 3	All assessed cases showed proficient mismatch repair (pMMR)
p53 expression pattern *(IHC only)* †	Overexpressed / No overexpression / Equivocal / Not assessed	2 / 3 / 1 / 4	IHC-based p53 protein expression pattern only
KRAS mutation status *(targeted Oncomine sequencing)* †	Mutated / No KRAS mutation detected	10 / 0	Pathogenic KRAS mutations were detected in all tumors, including codon 12 mutations in nine cases: p.Gly12Arg/G12R (*n* = 4), p.Gly12Val/G12V (*n* = 4), p.Gly12Asp/G12D (*n* = 1), and one codon 13 mutation: p.Gly13Asp/G13D (*n* = 1).
Additional molecular alterations from previous clinical molecular testing †	Present / Not reported	1 / 9	One case had previously reported ARID1A, CDKN2A, and CDKN2B alterations in the clinical record.
Histological tumor regression *(Evans grade)*	5 µM: 1 / 2a / 2b / 3	3 / 6 / 1 / 0	Low: 9 (grades 1 and 2a), High: 1 (2b)
	15 µM: 2a / 2b / 3	3 / 4 / 3	Low: 3 (2a), High: 7 (2b and 3)
Histological tumor regression *(CAP score)*	5 µM: 1 / 2 / 3	0 / 1 / 9	Low: 9 (score 3), High: 1 (score 2)
	15 µM: 1 / 2 / 3	2 / 6 / 2	Low: 2 (score 3), High: 8 (scores 1 and 2)

* According to the 8^th^ edition of the AJCC classification

** classified according to the 5^th^ edition of the World Health Organization (WHO) Classification of Tumors of the Digestive System, 2019

† MMR and p53 IHC findings were obtained from routine diagnostic assessment. Targeted Oncomine sequencing was performed on FFPE tumor tissue from all 10 cases in the ex vivo cohort. Previous clinical molecular testing was available from the clinical record in one case. Molecular findings were used for cohort characterization. p53 IHC expression pattern was recorded as reported in the diagnostic pathology assessment and used as an IHC-based descriptive variable

*Abbreviations: PDAC* Pancreatic ductal adenocarcinoma, *AJCC* American Joint Committee on Cancer, *TNM* Tumor–node–metastasis, *CAP* College of American Pathologists, *MMR* Mismatch repair, *pMMR* Proficient mismatch repair, *IHC* Immunohistochemistry, *L* Lymphatic invasion, *V* Venous invasion, *Pn* Perineural invasion, *R0/R1* Microscopic margin status

To assess whether selenite induced histological tumor-cell injury/regression in patient-derived tissue, matched slices were evaluated relative to the patient-matched treatment-control slice by a pancreatic specialist pathologist blinded to condition.

**Fig. 4:**
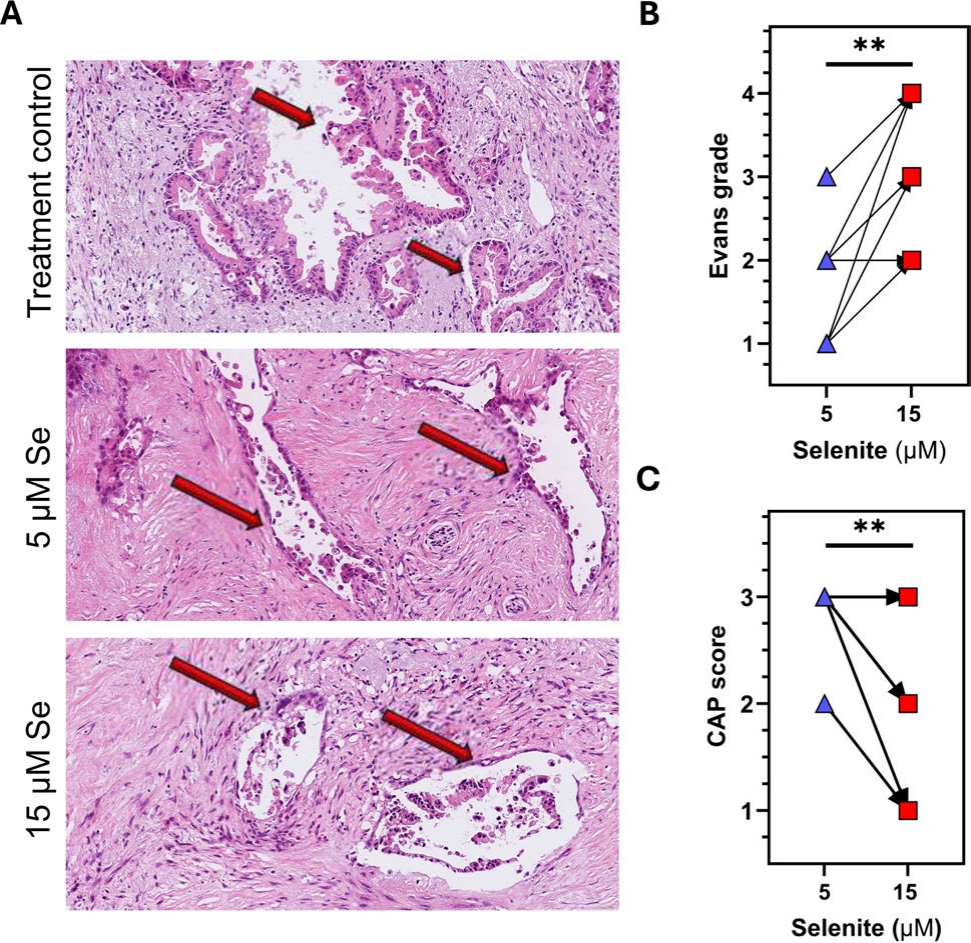
Dose-dependent histological tumor regression in paired ex vivo PDAC tissue slices after selenite. Matched PDAC tissue slices from *n* = 10 patients were treated ex vivo with sodium selenite at 5 µM or 15 µM and evaluated by a pancreatic specialist pathologist blinded to condition. **A** Representative H&E-stained sections of ex vivo PDAC tissue slices under treatment-control conditions and after 5 µM or 15 µM selenite treatment. Red arrows indicate representative tumor epithelial regions used to illustrate histological features considered during treatment-response assessment. Images were acquired at 63× magnification. Histological tumor regression was scored relative to the matched treatment-control tissue slice using (**B**) the Evans grading system and (**C**) the College of American Pathologists (CAP) system. For statistical testing, Evans grades were converted to an ordinal numeric scale (1 → 1, 2a → 2, 2b → 3, 3 → 4). CAP scores were analyzed as ordinal values. Lines connect patient-matched samples across doses. Histological tumor regression was significantly higher at 15 µM than at 5 µM for both scoring systems (Wilcoxon signed-rank test). **p < 0.01

Representative H&E images of treatment-control, 5 µM, and 15 µM selenite-treated tissue slices are shown in Fig. 4A. These images illustrate treatment-associated histological changes in tumor epithelium, with more pronounced damage/regression of the tumor epithelium at 15 µM compared with the patient-matched treatment-control condition.

Histological tumor regression showed a dose-dependent improvement upon selenite treatment (Fig. 4B, C; Table 2). At 5 µM, Evans grades were 1 (*n* = 3), 2a (*n* = 6), and 2b (*n* = 1), while CAP scores were predominantly 3 (*n* = 9) with one case scored as 2. At 15 µM, the distribution shifted toward higher response (Evans: 2a *n* = 3, 2b *n* = 4, 3 *n* = 3; CAP: 1 *n* = 2, 2 *n* = 6, 3 *n* = 2), with 7/10 cases classified as high response by Evans grading and 8/10 cases by CAP scoring. Paired comparisons confirmed significant differences between doses using both histological regression scoring systems. One case showed discordant classification between Evans and CAP scores (Table 2).

To explore sources of inter-patient variability in histological response, exploratory correlation analyses were performed within each dose level to assess whether available clinicopathological or molecular features were associated with histological treatment-response scores. At 5 µM, Evans grade correlated with TNM stage (*r* = 0.81, *p* = 0.0198). At 15 µM, regression correlated with age in both systems (Evans: *r* = 0.73, *p* = 0.0159; CAP: *r* = − 0.73, *p* = 0.016; lower CAP indicates better response). CAP score also correlated with tumor size (*r* = 0.65, *p* = 0.041), consistent with weaker histological response in larger tumors at 15 µM. Because all tumors carried pathogenic KRAS mutations, comparison between KRAS-mutant and KRAS-wild-type tumors was not possible. Exploratory stratification by KRAS variant type, p53 IHC pattern, and differentiation grade did not identify a consistent association with histological response. Given the small cohort size and the exploratory nature of these analyses, these associations should be regarded as hypothesis-generating.

Overall, these results show a dose-dependent increase in histological tumor-cell injury/regression, with most cases meeting the predefined high-response criteria at 15 µM. The exploratory association analyses did not indicate a clear relationship between histological response and KRAS variant type, p53 IHC pattern, or tumor differentiation grade in this small cohort.

#### Baseline EMT markers and culture-time effects

**Fig. 5:**
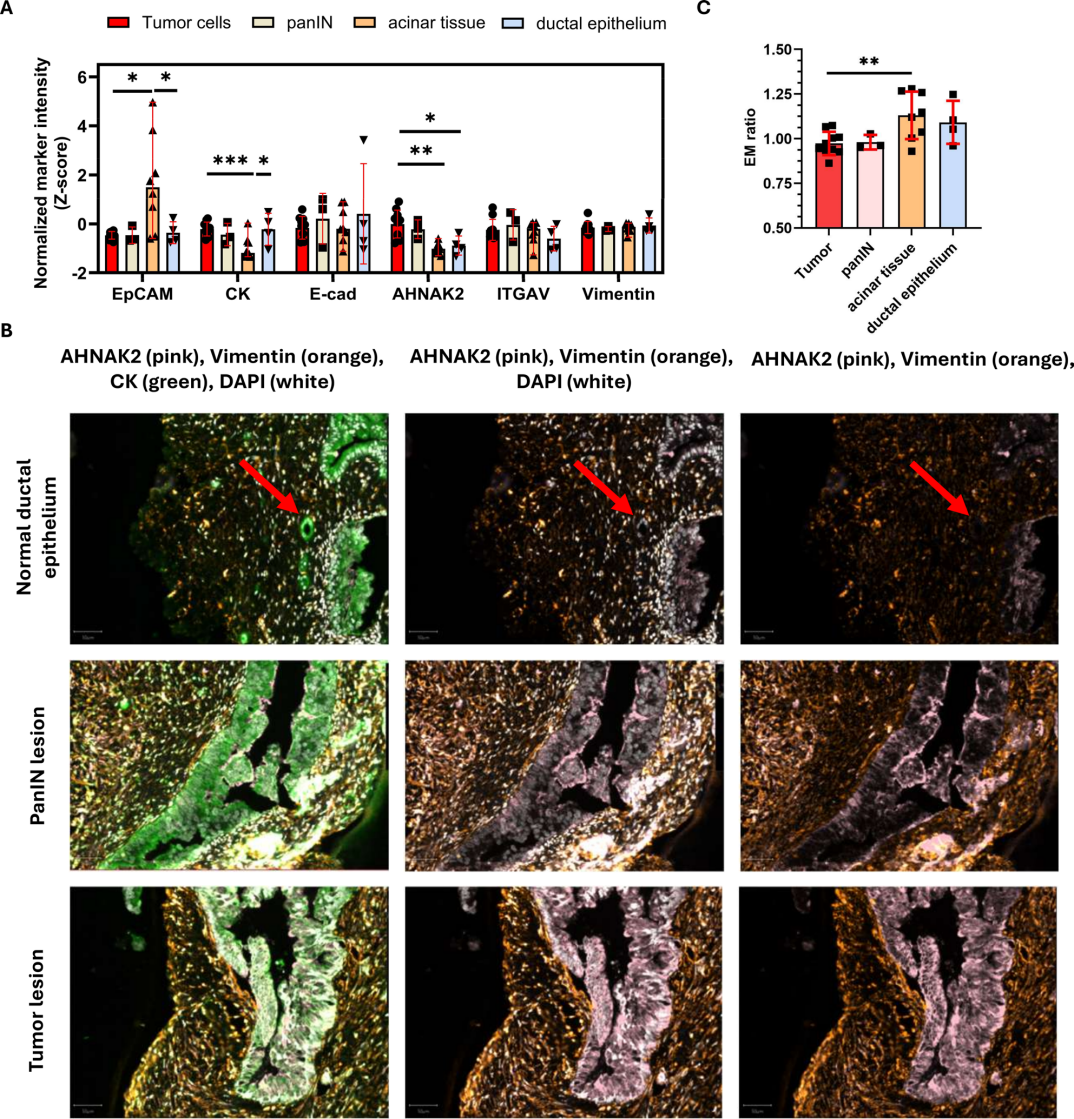
Baseline compartment-specific EMT marker patterns in ex vivo PDAC tissue slices. Multiplex immunofluorescence quantified epithelial (EpCAM, CK, E-cad) and mesenchymal (AHNAK2, ITGAV, Vimentin) markers in patient-derived PDAC tissue slices. **A** Baseline (0 h) marker expression across annotated compartments (tumor epithelium, PanIN, ductal epithelium, acinar tissue) from *n* = 10 patients. To mitigate technical variation within donors, values are presented as within-patient, marker-wise Z-scores as described in Methods. **B** Representative multiplex immunofluorescence images showing AHNAK2 (pink), Vimentin (orange), cytokeratin (CK; green), and DAPI (white) in normal ductal epithelium, PanIN, and tumor regions. The red arrow marks normal ductal epithelium. Images illustrate higher AHNAK2 signal in tumor compared with ductal and PanIN regions. Scale bar, 50 μm. **C** Baseline epithelial–mesenchymal (EM) ratio across compartments, calculated as the geometric mean of epithelial markers divided by the geometric mean of mesenchymal markers (higher values indicate a more epithelial phenotype). Bars show mean ± SD. Kruskal–Wallis with Dunn’s multiple comparisons test *p < 0.05, **p < 0.01, ***p < 0.001

Before assessing treatment effects, compartment-specific baseline marker expression and culture-time effects were examined to distinguish ex vivo adaptation from selenite-associated changes. Baseline (0 h) slices showed compartment-specific marker patterns (Fig. 5A). EpCAM was higher in acinar tissue than in tumor and ductal regions, whereas CK was higher in tumor and ducts than in acinar areas. AHNAK2 was highest in tumor compared with both acinar and ductal epithelium; values were slightly higher in PanIN than in ductal epithelium but reached statistical significance only in tumor (Fig. 5B). The EM ratio was higher in tumor than in acinar tissue, consistent with a more epithelial-like baseline state in tumor regions (Fig. 5C). PanIN and ductal epithelium were present in only a few cases (PanIN *n* = 3; ducts *n* = 4) and were under-represented at later timepoints, therefore, time-course and treatment comparisons focused on tumor and acinar compartments.

**Fig. 6:**
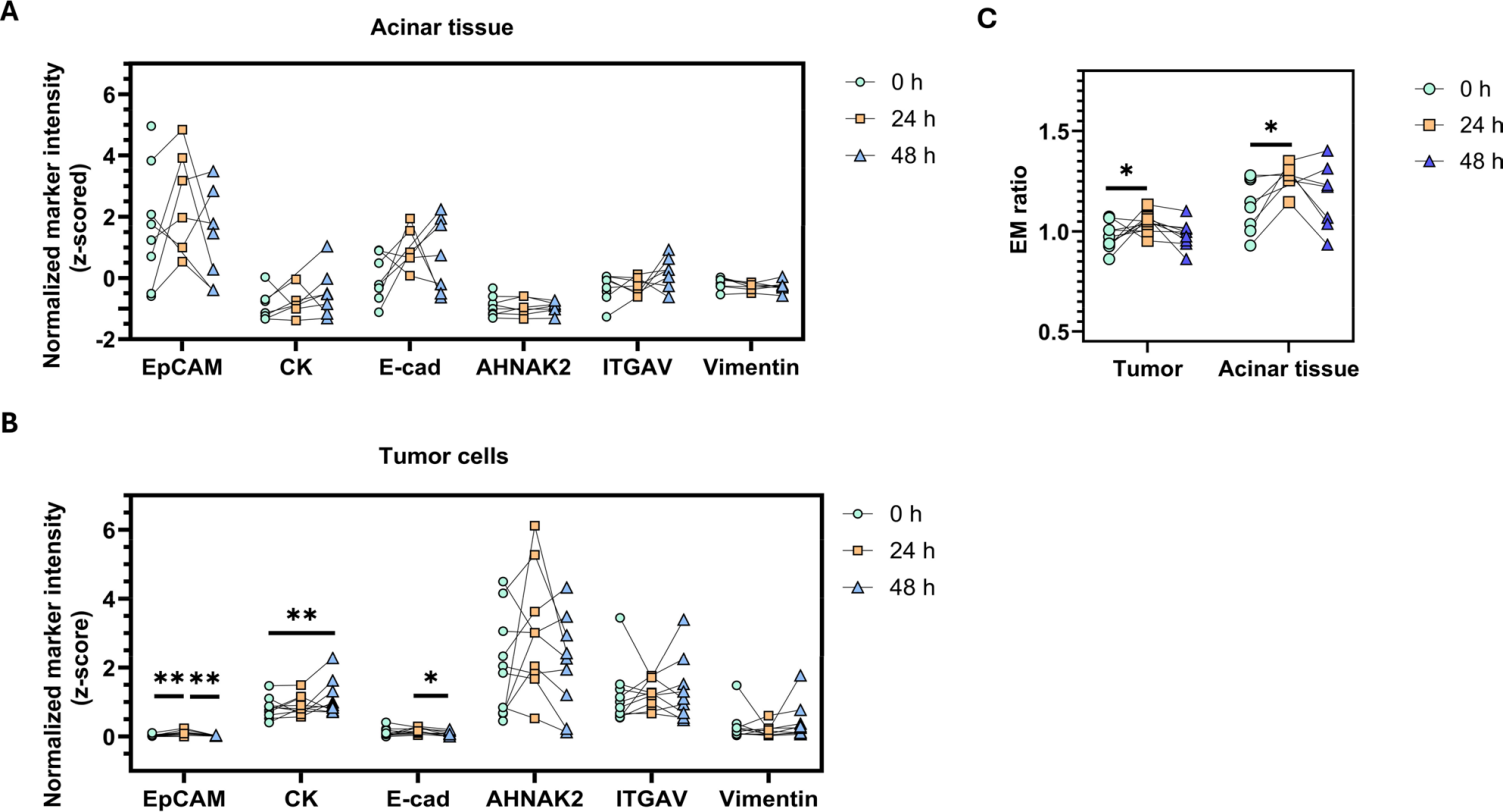
Culture-time effects on EMT markers in patient-matched ex vivo PDAC tissue slices. EMT marker intensities were quantified at 0 h (baseline), 24 h (culture control), and 48 h (untreated control) in (**A**) acinar and (**B**) tumor compartments from patient-derived PDAC tissue slices. Epithelial (EpCAM, CK, E-cad) and mesenchymal (AHNAK2, ITGAV, Vimentin) markers are shown for each timepoint. Points represent patient-matched samples and lines connect matched timepoints within donors. **C** Corresponding epithelial–mesenchymal (EM) ratio trajectories, calculated as the geometric mean of epithelial marker values divided by the geometric mean of mesenchymal marker values (higher values indicate a more epithelial phenotype), as defined in Methods. Time-course comparisons were performed within each compartment using a repeated-measures mixed-effects model with Tukey’s multiple-comparisons test. *p < 0.05, **p < 0.01

To assess culture-time effects, untreated slices were compared at 0 h, 24 h, and 48 h. EMT markers were stable in acinar tissue across timepoints (Fig. 6A). In tumor regions, EpCAM increased at 24 h versus baseline but returned to baseline by 48 h; CK was higher at 48 h versus 0 h, with no difference between 24 h and 48 h; E-cad was largely unchanged, showing only a slight decrease at 48 h versus 24 h. Mesenchymal markers remained stable at all timepoints in both compartments (Fig. 6B). The EM ratio mirrored these patterns, increasing from 0 h to 24 h without further change thereafter; 48 h values did not differ from baseline (Fig. 6C).

Together, these analyses defined the baseline and culture-time changes in the ex vivo model and supported the use of the 48 h untreated condition as the within-patient reference for subsequent treatment comparisons.

#### Compartment-specific EMT marker changes after selenite treatment

**Fig. 7:**
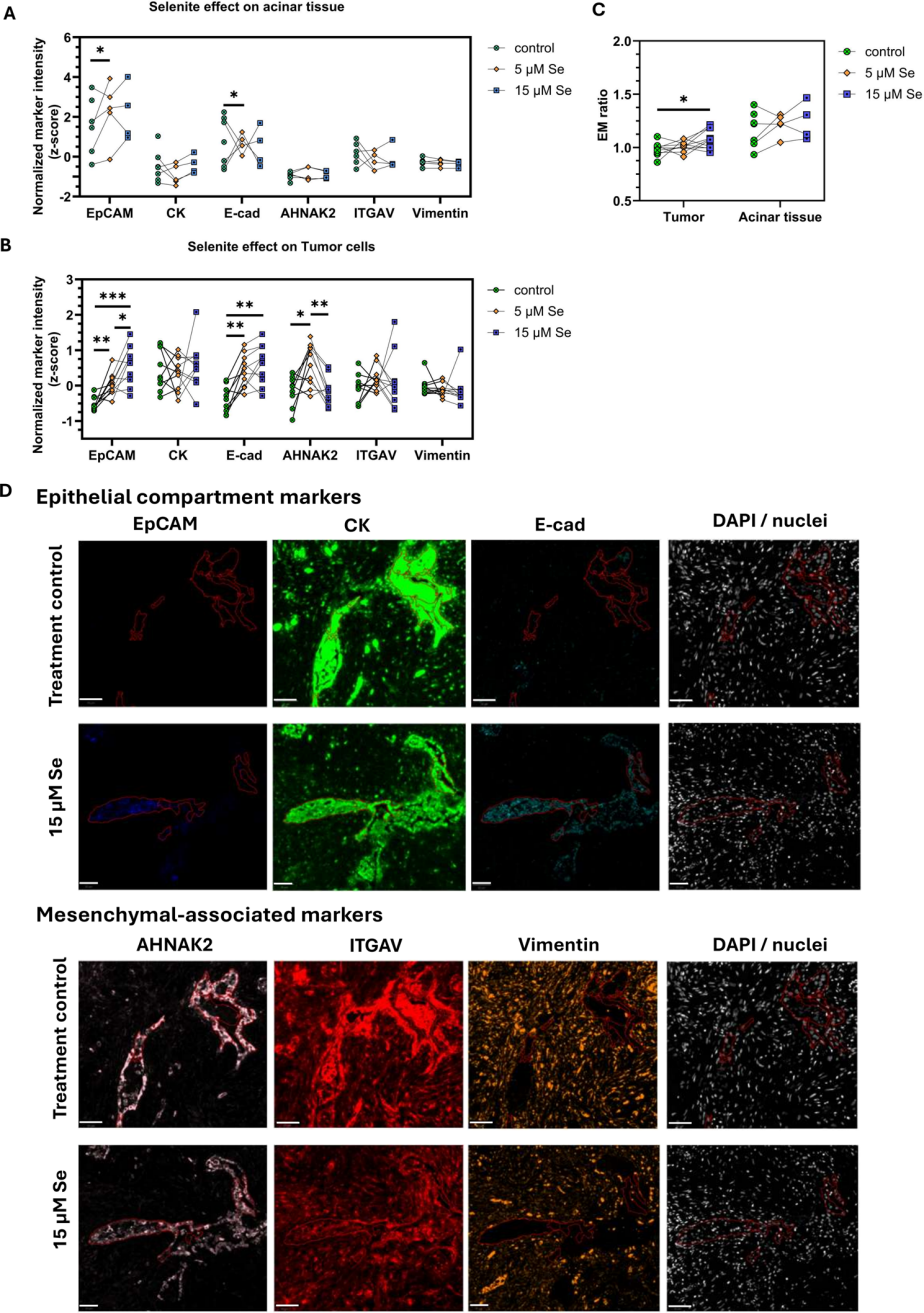
Selenite shifts compartment-resolved EMT marker patterns in ex vivo PDAC tissue slices. Within-patient, marker-wise normalized multiplex immunofluorescence intensities are shown for (**A**) acinar tissue and (**B**) tumor regions in patient-derived PDAC tissue-slice cultures (*n* = 10). Slices were analyzed at 48 h after culture with 0 µM (patient-matched untreated control), 5 µM, or 15 µM sodium selenite (treatment during 24–48 h). Points show patient-matched values; lines connect matched conditions within donors. **C** Epithelial–mesenchymal (EM) ratio in tumor and acinar compartments, calculated as the geometric mean of epithelial marker values (EpCAM, CK, E-cad) divided by the geometric mean of mesenchymal marker values (AHNAK2, ITGAV, Vimentin); higher values indicate a more epithelial phenotype. **A–C** Statistical comparisons were performed within each compartment using a repeated-measures mixed-effects model with Tukey’s multiple-comparisons test. *p < 0.05, **p < 0.01, ***p < 0.001. **D** Representative single-marker multiplex immunofluorescence images from patient-matched treatment-control and 15 µM selenite-treated tumor regions. Red outlines indicate pathologist-annotated tumor areas. Scale bar, 50 μm

Having established dose-dependent histological tumor regression, we next assessed whether selenite treatment was accompanied by compartment-specific shifts in EMT-marker expression. Analyses were restricted to tumor and acinar compartments in pathologist-annotated tissue sections (Fig. 7A–B) as ductal epithelium and PanIN lesions were sparse and further depleted by sequential sectioning, precluding robust statistical testing. In acinar tissue, EMT-marker expression was largely unchanged by selenite: EpCAM increased at 15 µM versus untreated control, and CK differed modestly between 5 µM and 15 µM, whereas E-cad, AHNAK2, ITGAV, and Vimentin showed no significant changes. Accordingly, the acinar EM ratio remained stable across conditions (Fig. 7C).

In tumor regions, selenite primarily modulated epithelial markers. EpCAM increased dose-dependently. E-cad was increased after both 5 µM and 15 µM selenite treatment compared with the treatment control, with no significant difference detected between the two selenite doses. AHNAK2 responded non-linearly, increasing at 5 µM and returning to baseline at 15 µM. Vimentin and ITGAV did not exhibit consistent treatment-associated changes. Consistent with these epithelial-leaning shifts, the tumor EM ratio was significantly increased at 15 µM (Fig. 7C). Representative multiplex IF images are shown in Fig. 7D.

In summary, the compartment-specific protein analysis showed that selenite-associated changes in tumor regions were most evident for epithelial markers, while mesenchymal markers showed no consistent treatment-associated change. Although histological tumor-cell injury/regression and tumor EM-ratio changes were both most evident at 15 µM, patient-level histological response scores did not show a robust association with treatment-induced tumor EM-ratio changes (Evans: ρ = −0.053, *p* = 0.891; CAP score: ρ = 0.129, *p* = 0.741; lower CAP indicates stronger response).

To further examine inter-patient variability in EMT-marker responses, within-patient Δ values (treated minus patient-matched untreated control) were calculated for each marker and the EM ratio, together with ΔΔ (15 µM − 5 µM) to capture dose-dependent changes. Treatment-induced changes in the tumor-compartment EM ratio were not consistently associated with sex, differentiation grade, vascular invasion, KRAS variant type, or p53 IHC pattern. Beyond the EM-ratio level, selected dose- and marker-specific associations were observed. ΔVimentin after 5 µM correlated positively with age (ρ = 0.75, *p* = 0.0199), whereas this association was not present at 15 µM. ΔCK after 5 µM correlated negatively with tumor size (ρ = −0.86, *p* = 0.0028), with no corresponding association at 15 µM. TNM stage correlated positively with ΔVimentin after 15 µM (ρ = 0.71, *p* = 0.033), and ΔΔ Vimentin also correlated positively with TNM stage (ρ = 0.67, *p* = 0.048), suggesting that higher-stage tumors showed an attenuated decrease in Vimentin after dose escalation from 5 to 15 µM. No comparable trends were detected for ITGAV or AHNAK2. Given the small cohort size, these exploratory analyses should be regarded as hypothesis-generating rather than predictive.

#### RNA-seq: culture effects and selenite response

Transcriptomic profiling in a subset of donors (*n* = 4) was performed to complement the compartment-resolved protein analysis with bulk gene-expression data, focusing on both culture-time adaptation and selenite-associated transcriptional changes.

##### Transcriptional changes associated with ex vivo culture duration

**Fig. 8:**
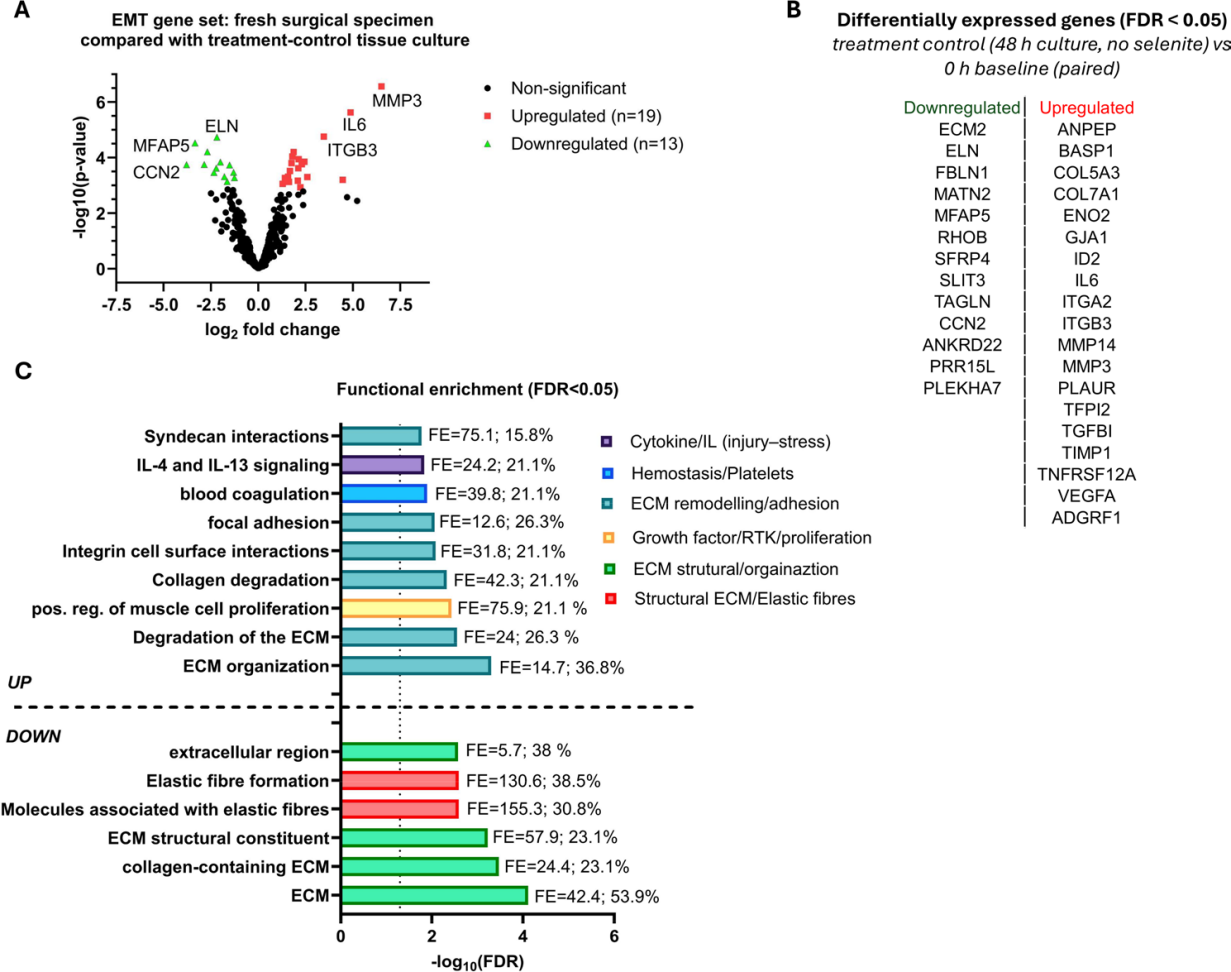
Ex vivo culture duration drives reproducible transcriptional adaptation in PDAC tissue slices. **A** Volcano plot of differential expression for 48 h treatment control versus 0 h baseline in ex vivo PDAC tissue-slice cultures (*n* = 4 donors) using edgeR with a paired design (patient as blocking factor). The x-axis shows log₂ fold change and the y-axis shows − log₁₀(p). Differential expression was defined by Benjamini–Hochberg FDR < 0.05 and |log₂FC| ≥ 0.58; genes meeting these criteria are highlighted by direction of change. **B** Summary of differentially expressed genes meeting FDR < 0.05 and log₂FC ≥ 0.58, grouped by direction of change. **C** Functional enrichment analysis of the differentially expressed gene sets using DAVID (v2024). Representative non-redundant GO and pathway terms are shown (BH-adjusted q ≤ 0.05). Bars are ordered by − log₁₀(FDR); labels indicate fold enrichment (FE) and the fraction of input genes mapping to each term. Full enrichment results are provided in Additional file 1: Table S2

To distinguish culture-time effects from treatment-associated changes, differential expression between fresh tissue (0 h) and the 48 h treatment controls was first characterized. Paired differential expression (edgeR; FDR < 0.05; |log₂FC| ≥ 0.58) comparing fresh tissue (0 h) with the 48 h treatment controls captured a reproducible ex vivo adaptation signature (Fig. 8A–B). Enrichment analysis indicated that up-regulated programs were dominated by extracellular matrix (ECM) remodeling and adhesion-related processes, including extracellular matrix organization, collagen degradation, integrin cell surface interactions, syndecan interactions, and focal adhesion (Fig. 8C). Representative genes contributing to these terms included COL5A3, COL7A1, ITGA2, ITGB3, MMP3, MMP14, TIMP1, TGFBI, PLAUR, and GJA1. Additional enriched annotations encompassed hemostasis/blood coagulation (ITGA2, ITGB3, PLAUR, TFPI2), interleukin signaling (IL-4/IL-13: TIMP1, IL6, MMP3, VEGFA), and growth factor–linked proliferation signatures (positive regulation of smooth muscle cell proliferation, ID2, ITGA2, ITGB3, IL6).

Down-regulated terms were enriched for structural ECM and elastic-fiber modules, including elastic fibre formation and molecules associated with elastic fibers (ELN, FBLN1, MATN2, MFAP5), as well as ECM structural organization–related categories such as extracellular matrix, collagen-containing ECM, ECM structural constituent, and extracellular region (CCN2, ELN, ECM2, FBLN1, MATN2, MFAP5, SFRP4). Collectively, these results indicate that the dominant transcriptional differences between 0 h and 48 h reflected culture-time adaptation, supporting the use of the patient-matched 48 h untreated condition as the reference for selenite-treatment effects.

##### Dose-dependent selenite effects on EMT-related transcription

**Fig. 9:**
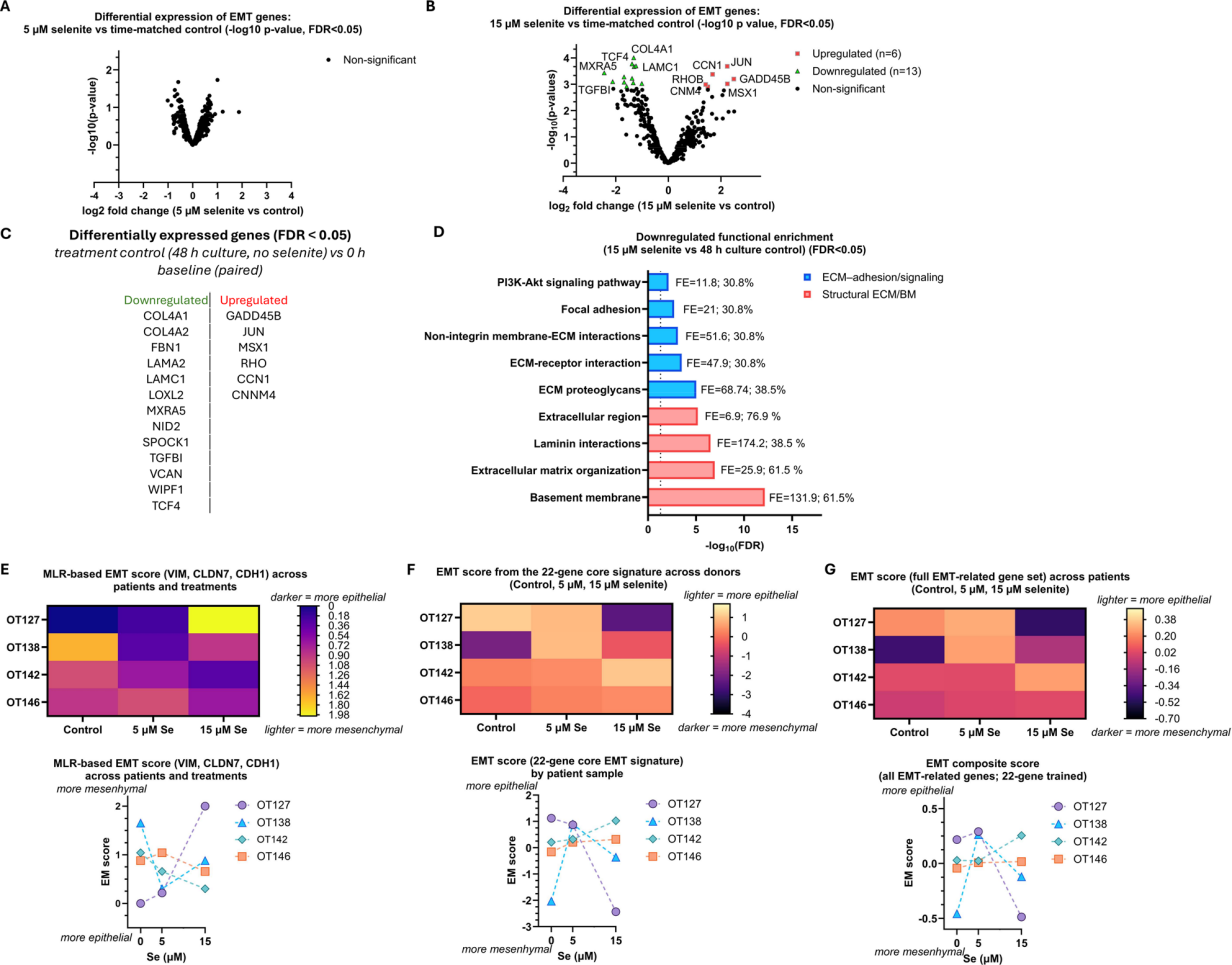
Dose-dependent selenite transcriptomic response and EMT scoring in ex vivo PDAC slices. **A**,** B** Volcano plots of differential expression versus the patient-matched 48 h treatment control in ex vivo PDAC tissue-slice cultures (*n* = 4 donors; OT127, OT138, OT142, OT146): (**A**) 5 µM and (**B**) 15 µM selenite (edgeR; paired model with patient as blocking factor; TMM normalization). Axes show log₂ fold change and − log₁₀(p-value); genes meeting Benjamini–Hochberg FDR < 0.05 and |log_2_FC| ≥ 0.58 are highlighted by direction (up, red; down, green; nonsignificant, black). **C** Differentially expressed genes at 15 µM (HGNC symbols), grouped by direction of change. **D** DAVID (v2024) functional enrichment of the 15 µM differentially expressed genes; representative non-redundant GO/pathway terms with BH-adjusted q ≤ 0.05 are shown. Bars are ordered by − log₁₀(FDR); labels indicate fold enrichment (FE) and the fraction of input genes annotated to each term. Full enrichment results are provided in Additional file 1: Table S3. **E–G** EMT scoring across donors for matched 48 h control, 5 µM, and 15 µM conditions (top, per-donor heatmaps; bottom, matched trajectories), computed as described in Methods. **E** MLR-style proxy (higher = more mesenchymal-like). **F** PDAC core 22-gene EM score (E − M; higher = more epithelial-like). **G** Extended 448-gene EM score (E − M; higher = more epithelial-like)

To control for culture-induced adaptation, selenite effects were analyzed relative to the patient-matched 48 h treatment control. Using a paired edgeR design (FDR < 0.05; |log₂FC| ≥ 0.58), no significant differential expression was detected at 5 µM within the curated EMT-related subset (Fig. 9A). 15 µM selenite elicited a compact response (Fig. 9B–C) comprising a small up-regulated set of immediate-early/stress-responsive genes and matrix-linked signaling modulators (GADD45B, JUN, MSX1, RHOB, CCN1, CNNM4) and a larger down-regulated set enriched for basement-membrane/ECM-associated genes (COL4A1, COL4A2, FBN1, LAMA2, LAMC1, LOXL2, MXRA5, NID2, SPOCK1, TGFBI, VCAN, WIPF1, TCF4) (Fig. 9A–C).

Consistent with the multiplex protein panel used, none of the corresponding transcripts reached differential-expression significance at 15 µM versus the paired 48 h control under the same thresholds (FDR < 0.05; |log₂FC| ≥ 0.58).

Enrichment analysis of the 15 µM down-regulated genes (DAVID v2024; BH FDR ≤ 0.05) highlighted basement-membrane/structural ECM and ECM–adhesion/signaling modules (Fig. 9D), including Basement membrane, ECM organization, Laminin interactions, ECM–receptor interaction, non-integrin membrane–ECM interactions, focal adhesion and PI3K-Akt signaling pathway. The up-regulated list was small (*n* = 6) and did not yield significant terms under the pre-specified ORA settings (Count ≥ 3; BH FDR ≤ 0.05). At the gene level, increases were confined to immediate-early/stress-responsive factors (JUN, GADD45B, RHOB) and matrix-linked signaling modulators (CCN1/CYR61, MSX1, CNNM4). Thus, at 15 µM, the transcriptional response was focused on attenuation of ECM/basement-membrane and adhesion-related programs, together with a limited stress-responsive up-regulated gene set.

To summarize treatment-associated EMT shifts, three complementary EMT metrics were applied (Fig. 9E–G). At 5 µM, the MLR metric indicated epithelial shifts in 2/4 donors and mesenchymal shifts in 2/4, whereas both panel-based metrics indicated epithelial shift in 3/4 donors (1/4 mesenchymal). At 15 µM, all three metrics indicated epithelial shift in 3/4 donors, with one donor shifting toward mesenchymal. Cross-metric concordance was greater at 15 µM than at 5 µM, with three of four donors showing an epithelial-leaning shift across all three scoring frameworks.

## Discussion

While the redox-disrupting and cytotoxic properties of selenite are well established [7, 10], its potential to modulate epithelial–mesenchymal features in pancreatic cancer has been less well explored. To our knowledge, this is the first integrated in vitro-ex vivo PDAC analysis of selenite-associated EMT modulation, spanning EMT-spectrum cell lines (± TGF-β) and patient-derived slices, and triangulating multiplex immunofluorescence with tumor-regression grading and paired RNA-seq in selected donors. A key strength of the ex vivo component is the within-patient paired slice-culture design, which enabled matched comparisons within each donor while preserving tumor architecture and stromal context, and allowed histological tumor regression and compartment-specific EMT-marker changes to be assessed in parallel.

In vitro selenite modulated EMT-associated features in a phenotype-contingent manner. The mesenchymal-biased PANC-1, consistent with its previously reported mesenchymal phenotype [46], showed only limited modulation, with no meaningful restoration of epithelial markers. This limited epithelial-marker response is also consistent with the concept that EMT reversibility is state-dependent, with partial or hybrid EMT states retaining greater plasticity than more stabilized mesenchymal states [47]. In keeping with this state-dependent pattern, the more epithelial-biased HPAF-II and Capan-2 lines exhibited clearer shifts along the EMT axis, including suppression of mesenchymal markers (notably ITGAV and Vimentin) and a concomitant increase in EM ratio.

These observations are directionally consistent with prior reports in other tumor types. In cholangiocarcinoma, a reduced N-cad/E-cad ratio was reported after selenite exposure, suggesting an epithelial-leaning shift [48]. Similarly, in renal cell carcinoma, selenite reduced MMP-9 and increased E-cad in vitro and in vivo [14]. However, these studies did not evaluate baseline EMT status [14, 48], limiting conclusions about state-dependent responsiveness. In our study, E-cad was not consistently increased across lines, yet the coordinated reduction of mesenchymal markers together with epithelial marker reinforcement, most evident in HPAF-II and Capan-2, supports phenotypic EMT modulation by selenite. Collectively, our data extend previous observations by suggesting that selenite-associated shifts in EMT-related marker patterns depend on intrinsic cellular state, with mesenchymal-like cells showing lower responsiveness and epithelial-biased lines showing more consistent epithelial reinforcement.

To assess whether TGF-β pretreatment alters the EMT-modulatory effect of selenite, we observed distinct responses across the cell lines. In PANC-1, TGF-β pretreatment further constrained epithelial marker responses to selenite and did not produce a meaningful increase in EM ratio. In contrast, HPAF-II and Capan-2 retained epithelial-promoting responses to selenite after TGF-β exposure, although the magnitude differed; in Capan-2, this was evident only at the higher selenite dose, suggesting reduced sensitivity at the lower dose after partial EMT induction. Notably, in HPAF-II, TGF-β alone induced a clearer EMT shift even without selenite treatment. Although the underlying mechanisms were not addressed here, these differences may reflect cell line–specific plasticity or variation in responses to EMT-inducing stimuli. Overall, our data indicate that selenite more consistently shifted the EM balance in contexts where mesenchymal commitment remained partial and potentially reversible.

To capture PDAC complexity and microenvironmental context [30, 49, 50], we used patient-derived ex vivo tissue-slice cultures. EMT marker expression in largely untreated samples closely mirrored the original tumor tissue, indicating that surgical stress and early ex vivo adaptation did not substantially alter EM features. Tumor regions showed a transient increase in epithelial markers at 24 h, partly declined by 48 h, suggesting an early adaptation phase. CK expression rose only at 48 h, consistent with delayed cell–cell contact reorganization. These temporal shifts were confined to tumor regions while acinar tissue remained stable, underscoring baseline variability and greater stress sensitivity/plasticity of tumor cells under ex vivo conditions when interpreting treatment effects. Transcriptomic profiling of the 0 h vs. 48 h controls indicates that the dominant signature reflects culture-time adaptation rather than EMT reprogramming per se, with enrichment for ECM/adhesion remodeling and reduced structural ECM/elastic-fiber modules. This interpretation is consistent with prior slice-culture studies reporting conserved injury/wound-healing and ECM-remodeling programs over short-term culture [50, 51].

Regarding the effect of selenite on EMT features in ex vivo PDAC slices, we observed patterns partly consistent with the in vitro data: selenite promoted a more epithelial phenotype within tumor epithelium, most clearly through upregulation of epithelial markers (EpCAM, E-cad), while mesenchymal markers showed no uniform suppression. Thus, in this setting EMT modulation appeared to act primarily via reinforcement of epithelial features rather than broad inhibition of the mesenchymal program. This compartment-specific pattern is consistent with the established concept of tumor redox vulnerability, whereby malignant cells operating under elevated basal oxidative stress may be more susceptible to additional redox disruption than non-malignant epithelial cells [7, 10]. Although normal ductal epithelium was too sparsely represented for robust quantitative analysis, preserved non-malignant structures did not show the same extent of treatment-associated histological changes as tumor regions. Across readouts, the response was dose-dependent [9, 10, 52]. Histologically, Evans and CAP grading indicated significantly stronger histological tumor regression at 15 µM than at 5 µM relative to patient-matched controls, and multiplex IF showed a significant increase in tumor EM ratio at 15 µM, consistent with an epithelial-shifting effect at higher dose. However, the magnitude of histological response was not robustly associated with treatment-induced tumor EM-ratio changes at the individual-donor level, indicating that these readouts may occur in parallel within the same treatment context rather than being directly proportional to one another. Transcriptomically, paired analysis also followed this dose-dependent pattern: 5 µM yielded no significant EMT-related DE versus the 48-h control, whereas 15 µM induced a compact response with limited upregulation of immediate-early/stress-responsive genes and matrix-linked signaling modulators, alongside broader downregulation enriched for basement-membrane/ECM components. Rather than a canonical MET program, the 15 µM signature was characterized by attenuation of ECM/basement-membrane and adhesion-related modules, including downregulation of genes such as TGFBI, a TGF-β–inducible glycoprotein implicated in desmoplastic EMT-supporting programs in PDAC. This transcriptional pattern may contribute to, or accompany, the epithelial-leaning shifts observed at the protein level. Collectively, the convergent readouts support a model in which higher-dose selenite was associated with reinforcement of epithelial features in malignant compartments while transcriptionally dampening matrix/adhesion programs.

We applied three complementary EMT metrics: a MLR surrogate, a PDAC-tailored 22-gene panel (core-EMT), and a genome-wide score, to capture distinct EMT facets and mitigate method-specific bias [39, 41, 43]. Across donors, all three metrics supported a dose-dependent shift toward epithelial features, with heterogeneous responses at 5 µM (MLR: 2/4 epithelial vs. 2/4 mesenchymal; panel-based: 3/4 epithelial) and a more consistent epithelial shift at 15 µM (3/4 donors; 1/4 mesenchymal-leaning). The partial cross-metric discordance at 5 µM is expected given that the MLR emphasizes polarity/adhesion contrasts, whereas the PDAC panel and its genome-wide extension reflect broader epithelial programs. Notably, the stronger genome-wide shift at 15 µM is consistent with the transcriptomic attenuation of ECM/basement-membrane and adhesion modules at this dose, in line with the epithelial-leaning protein readouts. Overall, these metrics support dose-dependent epithelial reinforcement while highlighting donor-specific variability.

Donor-specific divergence may partly reflect tissue composition. Because PDAC slices are highly desmoplastic, preferential injury of malignant epithelium by selenite [10] may reduce epithelial signal in low–tumor-fraction specimens and cause bulk EMT scores to be dominated by relatively spared stroma, creating an apparent mesenchymal shift despite histological tumor regression. Consistent with this, our outlier donor had comparable Evans/CAP regression and no distinguishing clinical features, while multiplex IF within tumor ROIs still indicated an epithelial shift at 15 µM, underscoring potential confounding by tumor cellularity/stromal content.

To explore whether inter-patient variability in selenite-associated EMT shifts was related to available clinical, histopathological, or molecular features, we correlated treatment-induced changes with clinicopathological variables and available molecular data. These associations are exploratory and should be interpreted cautiously given the small cohort size. Treatment-induced changes in the tumor-compartment EM ratio were not consistently associated with sex, differentiation grade, vascular invasion, KRAS variant type, or p53 IHC pattern. Among individual markers, several dose- and marker-specific associations emerged. At 5 µM, older patients tended to retain higher ΔVimentin, and larger tumors showed a smaller ΔCK increase, consistent with heterogeneous low-dose responses. At 15 µM, variability partly narrowed, yet TNM stage remained informative: more advanced tumors showed a less pronounced decrease in Vimentin after dose escalation from 5 to 15 µM, suggesting an attenuated decrease in this mesenchymal-associated marker at higher stage. This pattern is consistent with reduced suppression of selected mesenchymal-associated features in higher-stage tumors, aligning with our in vitro observation that the mesenchymal-biased PANC-1 was less responsive than epithelial-leaning lines. Given the small cohort (*n* = 10), these clinicopathological and molecular correlations should be regarded as hypothesis-generating and motivate validation in larger molecularly annotated cohorts.

A few limitations define the scope of the current work. Progressive tissue loss during sectioning led to underrepresentation of PanIN and ductal compartments, limiting statistical assessment in these regions. The cohort was limited to surgically resectable PDAC, and bulk RNA-seq was available from four donors. Because bulk RNA-seq is composition-sensitive, these data may be influenced by tumor–stroma ratios, although this limitation was partly mitigated by the paired design and tumor ROI–restricted multiplex immunofluorescence. The present study assessed selenite-associated tumor response using established histological regression scoring together with compartment-resolved EMT-marker analysis and paired transcriptomics. However, assigning these effects to individual regulated cell-death pathways, or separating phenotypic modulation of surviving tumor cells from selective loss of specific tumor-cell states, will require dedicated mechanistic follow-up studies. Despite these constraints, this study provides a clinically proximal, compartment-resolved view of how redox disruption can be accompanied by EMT-associated phenotype shifts in PDAC. By integrating baseline state profiling in cell lines with patient-derived tissue slices, and by aligning multiplex protein readouts with histological regression and paired transcriptomics, we identify a dose- and context-dependent pattern in which selenite predominantly affected malignant regions and was associated with reinforcement of epithelial features while dampening ECM/adhesion programs at higher dose. This integrated framework helps bridge preclinical redox biology with clinically observed variability in selenite response and supports continued translational evaluation of selenite in PDAC using model systems that preserve tumor architecture and stromal context.

## Conclusions

In summary, selenite induced dose- and context-dependent shifts in EMT-associated features in PDAC models. In vitro, epithelial-biased cell lines showed the clearest epithelial reinforcement and suppression of selected mesenchymal markers, whereas a mesenchymal-biased line was less responsive. In patient-derived PDAC tissue slices, selenite-associated EMT-marker changes were most evident in malignant regions, where higher-dose selenite was associated with stronger histological tumor regression and increased tumor EM ratio. Paired transcriptomics indicated that, beyond culture-time adaptation, higher-dose selenite was accompanied by focused downregulation of basement-membrane/ECM and adhesion-related programs rather than a canonical MET transcriptional switch. Together, these data support further translational evaluation of selenite in PDAC using composition-aware, compartment-resolved models and motivate efforts to identify molecular and histopathological response modifiers of dose-dependent EMT-associated responses.

## Supplementary Information

Supplementary Material 1: Table S1. Curated EMT-related 448-gene reference list with source annotations. Table S2. Functional enrichment for differential expression associated with ex vivo culture (48 h control vs 0 h baseline). Table S3. Functional enrichment for differential expression after 15 µM selenite treatment (15 µM vs paired 48 h control).

## Data Availability

RNA-seq data generated in this study have been deposited in the Gene Expression Omnibus (GEO) under accession GSE315148 (samples GSM9422154–GSM9422169).

## Abbreviations

PDAC: Pancreatic ductal adenocarcinoma
Se: Sodium selenite
EMT: Epithelial–mesenchymal transition
EpCAM: Epithelial cell adhesion molecule
CK: Cytokeratin
E-cad: E-cadherin
AHNAK2: AHNAK nucleoprotein 2
ITGAV: Integrin subunit alpha V
TGF-β: Transforming growth factor–β
CAP: College of American Pathologists
RNA-seq: RNA sequencing
ECM: Extracellular matrix
ROS: Reactive oxygen species
FOLFIRINOX: Fluorouracil, leucovorin, irinotecan, and oxaliplatin
MET: Mesenchymal–epithelial transition
EM: Epithelial-mesenchymal
AJCC: American Joint Committee on Cancer
FFPE: Formalin-fixed, paraffin-embedded
H&E: Hematoxylin and eosin
PanIN: Pancreatic intraepithelial neoplasia
ATCC: American Type Culture Collection
FBS: Fetal bovine serum
IC₂₅: Inhibitory concentration reducing viability by 25%
IC₅₀: Inhibitory concentration reducing viability by 50%
DNA: Deoxyribonucleic acid
IF: Immunofluorescence
IHC: Immunohistochemistry
DAPI: 4′,6-diamidino-2-phenylindole
SD: Standard deviation
HGNC: HUGO Gene Nomenclature Committee
DE: Differential expression
CPM: Counts per million
MLR: Multinomial logistic regression
DAVID: Database for Annotation, Visualization and Integrated Discovery
GO: Gene Ontology
KEGG: Kyoto Encyclopedia of Genes and Genomes
FDR: False discovery rate
BH: Benjamini–Hochberg
FE: Fold enrichment
ORA: Over-representation analysis
MMR: Mismatch repair
pMMR: Proficient mismatch repair
WHO: World Health Organization
GEO: Gene Expression Omnibus
TMM: Trimmed mean of M-values
