# Supplementary material for: Selenite modulates phenotype-dependent epithelial–mesenchymal plasticity in pancreatic ductal adenocarcinoma: integrated in vitro analyses and patient-derived ex vivo tissue-slice cultures
Source: J Exp Clin Cancer Res. 2026 Jul 13;45:160. doi: 10.1186/s13046-026-03778-4 (PMC13366627; doi:10.1186/s13046-026-03778-4)
Supplement: Supplementary file 1 — Supplementary Material 1: Table S1. Curated EMT-related 448-gene reference list with source annotations. Table S2. Functional enrichment for differential expression associated with ex vivo culture (48 h control vs 0 h baseline). Table S3. Functional enrichment for differential expression after 15 µM selenite treatment (15 µM vs paired 48 h control). [file 13046_2026_3778_MOESM1_ESM.docx]

**Supplementary Tables**

**Table S1. Curated EMT-related 448-gene reference list with source annotations.** EMT-related genes (HGNC symbols) were assembled by merging EMTome-curated gene sets [39], including the MSigDB Hallmark EMT gene set [40], the 22-gene PDAC core EMT panel [41], and a pan-cancer EMT signature [42], and by adding genes corresponding to the multiplex protein markers quantified in this study (EPCAM, CDH1, VIM, ITGAV, AHNAK2, and keratins represented by the panCK antibody: KRT1–KRT8, KRT10, KRT14–KRT16, KRT19). Superscript source codes indicate the originating list(s): ¹ MSigDB Hallmark EMT, ² Aiello et al. PDAC core panel, ³ Koplev et al. pan-cancer signature, ⁴ protein marker panel used in the present study. Gene symbols were harmonized to HGNC and filtered to remove non-coding entries, deprecated symbols/synonyms, and duplicates, yielding 448 unique genes.

| ***Gene*** *(HGNC symbol)* | | | | | | | | |
| --- | --- | --- | --- | --- | --- | --- | --- | --- |
| ABI3BP ^1^ | EFEMP2 ^1^ | LAMC2 ^1, 3^ | SAT1 ^1^ | EPCAM ^2, 3, 4^ | C1orf210 ^3^ | BAIAP2L1 ^3^ | PAK6 ^3^ | USP43 ^3^ |
| ACTA2 ^1^ | ELN ^1^ | LGALS1 ^1^ | SCG2 ^1^ | ESRP1 ^2^ | JUP ^3^ | ERBB3 ^3^ | TMEM45B ^3^ | IGSF9 ^3^ |
| ADAM12 ^1^ | EMP3 ^1^ | LOX ^1^ | SDC1 ^1^ | KRT7 ^2^ | C19orf21 (MISP) ^3^ | ATP2C2 ^3^ | ENPP5 ^3^ | AIM1L (CRYBG2) ^3^ |
| ANPEP ^1^ | ENO2 ^1^ | LOXL1 ^1^ | SDC4 ^1, 3^ | KRT8 ^2^ | OVOL2 ^3^ | CMTM4 ^3^ | CD46 ^3^ | ITPKC ^3^ |
| APLP1 ^1^ | FAP ^1, 2^ | LOXL2 ^1^ | SERPINE1 ^1^ | KRT18 ^2^ | INADL (PATJ) ^3^ | MYO5B ^3^ | CAMSAP3 ^3^ | PWWP2B ^3^ |
| AREG ^1^ | FAS ^1^ | LRP1 ^1^ | SERPINE2 ^1^ | KRT19 ^2, 3, 4^ | C6orf132 ^3^ | ADAP1 ^3^ | KIAA1671 ^3^ | GPR56 ^3^ |
| BASP1 ^1^ | FBLN1 ^1^ | LRRC15 ^1^ | SERPINH1 ^1^ | KRT20 ^2^ | CNKSR1 ^3^ | PLEKHG6 ^3^ | TMPRSS4 ^3^ | FRK ^3^ |
| BDNF ^1^ | FBLN2 ^1^ | LUM ^1, 2^ | SFRP1 ^1^ | MUC1 ^2, 3^ | RBM47 ^3^ | DSG2 ^3^ | ARRDC1 ^3^ | FAM83F ^3^ |
| BGN ^1^ | FBLN5 ^1^ | MAGEE1 ^1^ | SFRP4 ^1^ | CDH1 ^2, 3, 4^ | EPHA1 ^3^ | HOOK2 ^3^ | COBL ^3^ | CNNM4 ^3^ |
| BMP1 ^1^ | FBN1 ^1^ | MATN2 ^1^ | SGCB ^1^ | CLDN4 ^2, 3^ | DSC2 ^3^ | LIPH ^3^ | TPD52L1 ^3^ | GPR110 (ADGRF1) ^3^ |
| CADM1 ^1^ | FBN2 ^1^ | MATN3 ^1^ | SGCD ^1^ | GLI1 ^2^ | PKP3 ^3^ | VWA1 ^3^ | EHF ^3^ | PPAP2C  (PLPP2) ^3^ |
| CALD1 ^1^ | FERMT2 ^1^ | MCM7 ^1^ | SGCG ^1^ | PALLD ^2^ | TMEM30B ^3^ | SH3YL1 ^3^ | STYK1 ^3^ | TINAGL1 ^3^ |
| CALU ^1^ | FGF2 ^1^ | MEST ^1^ | SLC6A8 ^1^ | PDPN ^2^ | CGN ^3^ | ZNF165 ^3^ | LAMA5 ^3^ | S100A16 ^3^ |
| CAP2 ^1^ | FLNA ^1^ | MFAP5 ^1^ | SLIT2 ^1^ | CLDN2 ^2^ | C1orf116 ^3^ | AP1M2 ^3^ | PRR15L ^3^ | SULT2B1 ^3^ |
| CAPG ^1^ | FMOD ^1^ | MGP ^1^ | SLIT3 ^1^ | DDR2 ^2^ | GALNT3 ^3^ | CLDN3 ^3^ | TMC5 ^3^ | LCN2 ^3^ |
| CD44 ^1^ | FN1 ^1^ | MMP1 ^1^ | SNAI2 ^1^ | F11R ^3^ | ARHGEF16 ^3^ | PERP ^3^ | KCNK1 ^3^ | PRKCZ ^3^ |
| CD59 ^1^ | FOXC2 ^1^ | MMP14 ^1^ | SNTB1 ^1^ | ESRP2 ^3^ | DDR1 ^3^ | ELMO3 ^3^ | FUT1 ^3^ | FHDC1 ^3^ |
| CDH11 ^1, 2^ | FSTL1 ^1^ | MMP2 ^1^ | SPARC ^1, 2^ | CLDN7 ^3^ | TSPAN15 ^3^ | SHROOM3 ^3^ | SLPI ^3^ | RECK ^3^ |
| CDH2 ^1^ | FSTL3 ^1^ | MMP3 ^1^ | SPOCK1 ^1^ | MARVELD2 ^3^ | RHOD ^3^ | STAP2 ^3^ | LYPD3 ^3^ | CEP85L ^3^ |
| CDH6 ^1^ | FUCA1 | MSX1 ^1^ | SPP1 ^1^ | MARVELD3 ^3^ | MST1R ^3^ | PRR15 ^3^ | C2orf55 (CRACDL) ^3^ | FAM216A ^3^ |
| COL11A1 ^1^ | FZD8 ^1^ | MXRA5 ^1^ | TAGLN ^1^ | MPZL2 ^3^ | GRTP1 ^3^ | PLS1 ^3^ | ATP8B1 ^3^ | PDXP ^3^ |
| COL12A1 ^1^ | GADD45A ^1^ | MYL9 ^1^ | TFPI2 ^1^ | CRB3 ^3^ | KLF5 ^3^ | SLC52A3 ^3^ | GRHL3 ^3^ | FAM55C (NXPE3) ^3^ |
| COL16A1 ^1^ | GADD45B ^1^ | MYLK ^1^ | TGFB1 ^1^ | MAL2 ^3^ | ARHGEF5 ^3^ | ANXA9 ^3^ | ALS2CL ^3^ | SEPT6 (SEPTIN6) ^3^ |
| COL1A1 ^1^ | GAS1 ^1^ | NID2 ^1^ | TGFBI ^1^ | CDS1 ^3^ | EPS8L1 ^3^ | AGR2 ^3^ | KLC3 ^3^ | ANKRD44 ^3^ |
| COL1A2 ^1^ | GEM ^1^ | NNMT ^1^ | TGFBR3 ^1^ | LLGL2 ^3^ | SFN ^3^ | RAB17 ^3^ | FXYD3 ^3^ | TCF4 ^3^ |
| COL3A1 ^1^ | GJA1 ^1^ | NOTCH2 ^1^ | TGM2 ^1^ | SPINT1 ^3^ | CCDC64B ^3^ | PRRG4 ^3^ | TMEM54 ^3^ | IFFO1 ^3^ |
| COL4A1 ^1^ | GLIPR1 ^1^ | NT5E ^1^ | THBS1 ^1^ | DSP ^3^ | TSPAN1 ^3^ | RASEF ^3^ | TTC39A ^3^ | SACS ^3^ |
| COL4A2 ^1^ | GPC1 ^1^ | NTM ^1^ | THBS2 ^1^ | FAM83H ^3^ | CXCL16 ^3^ | MAPK13 ^3^ | STX19 ^3^ | CCDC88A ^3^ |
| COL5A1 ^1^ | GPX7 ^1^ | OXTR ^1^ | THY1 ^1^ | ELF3 ^3^ | TJP3 ^3^ | C19orf46 (SYNE4) ^3^ | EPB41L1 ^3^ | ST3GAL2 ^3^ |
| COL5A2 ^1^ | GREM1 ^1^ | PCOLCE ^1^ | TIMP1 ^1^ | SPINT2 ^3^ | CBLC ^3^ | ITGB4 ^3^ | PLEKHG3 ^3^ | SH2B3 ^3^ |
| COL5A3 ^1^ | HTRA1 ^1^ | PCOLCE2 ^1^ | TIMP3 ^1^ | TMC4 ^3^ | B3GNT3 ^3^ | FAM110C ^3^ | PTPN3 ^3^ | SLC35B4 ^3^ |
| COL6A2 ^1^ | ID2 ^1^ | PDGFRB ^1, 2^ | TNC ^1, 2^ | PPL ^3^ | TMEM184A ^3^ | FGFBP1 ^3^ | PLEKHA6 ^3^ | FBXO43 ^3^ |
| COL6A3 ^1^ | IGFBP2 ^1^ | PDLIM4 ^1^ | TNFAIP3 ^1^ | SCNN1A ^3^ | GRHL1 ^3^ | CYB561 ^3^ | RAB20 ^3^ | AP1S2 ^3^ |
| COL7A1 ^1^ | IGFBP3 ^1^ | PFN2 ^1^ | TNFRSF11B ^1^ | LAD1 ^3^ | RAB25 ^3^ | FOXA1 ^3^ | PROM2 ^3^ | ZEB2 ^3^ |
| COL8A2 ^1^ | IGFBP4 ^1^ | PLAUR ^1^ | TNFRSF12A ^1^ | LSR ^3^ | PRRG2 ^3^ | EPS8L2 ^3^ | RNF39 ^3^ | STARD9 ^3^ |
| COMP ^1^ | IL15 ^1^ | PLOD1 ^1^ | TPM1 ^1^ | TC2N ^3^ | MYH14 ^3^ | GJB3 ^3^ | F2RL1 ^3^ | AP1M1 ^3^ |
| COPA ^1^ | IL32 ^1^ | PLOD2 ^1^ | TPM2 ^1^ | MAP7 ^3^ | TACSTD2 ^3^ | PVRL4 (NECTIN4) ^3^ | KRTCAP3 ^3^ | AHNAK2 ^4^ |
| CRLF1 ^1^ | IL6 ^1^ | PLOD3 ^1^ | TPM4 ^1^ | C1orf172 ^3^ | MYO6 ^3^ | ANKRD22 ^3^ | SMPDL3B ^3^ | KRT10 ^4^ |
| CTHRC1 ^1^ | INHBA ^1^ | PMEPA1 ^1^ | VCAM1 ^1^ | IRF6 ^3^ | OVOL1 ^3^ | SERINC2 ^3^ | STEAP4 ^3^ | KRT14 ^4^ |
| CXCL1 ^1^ | ITGA2 ^1^ | PMP22 ^1^ | VCAN ^1^ | STARD10 ^3^ | MYO5C ^3^ | EPB41L4B ^3^ | PLEKHA7 ^3^ | KRT15 ^4^ |
| CXCL12 ^1^ | ITGA5 ^1^ | POSTN ^1, 2^ | VEGFA ^1^ | PRSS22 ^3^ | LRRC1 ^3^ | FAM83B ^3^ | GSTO2 ^3^ | KRT16 ^4^ |
| CXCL6 ^1^ | ITGAV ^1, 4^ | PPIB ^1^ | VEGFC ^1^ | CDH3 ^3^ | SLC44A3 ^3^ | RHPN2 ^3^ | HDHD3 ^3^ | KRT1 ^4^ |
| DAB2 ^1^ | ITGB1 ^1^ | PRRX1 ^1^ | VIM ^1, 4^ | S100A14 ^3^ | BSPRY ^3^ | P2RY2 ^3^ | MPP7 ^3^ | KRT2 ^4^ |
| DCN ^1^ | ITGB3 ^1^ | PRSS2 ^1^ | WIPF1 ^1^ | EPN3 ^3^ | EFNA1 ^3^ | TNK1 ^3^ | C19orf33 ^3^ | KRT3 ^4^ |
| DKK1 ^1^ | ITGB5 ^1^ | PTHLH ^1^ | WNT5A ^1^ | CHMP4C ^3^ | GRB7 ^3^ | ITGB6 ^3^ | WWC1 ^3^ | KRT4 ^4^ |
| DPYSL3 ^1^ | JUN ^1^ | PTX3 ^1^ | CCN2 ^1^ | GRHL2 ^3^ | EVPL ^3^ | S100P ^3^ | VGLL1 ^3^ | KRT5 ^4^ |
| DST ^1^ | LAMA1 ^1^ | PVR ^1^ | CCN1 ^1^ | KIAA1522 ^3^ | GOLT1A ^3^ | ANXA3 ^3^ | PPP1R13L ^3^ | KRT6 ^4^ |
| ECM1 ^1^ | LAMA2 ^1^ | QSOX1 ^1^ | COLGALT1 ^1^ | TMEM125 ^3^ | FA2H ^3^ | SERPINB5 ^3^ | SCEL ^3^ | ZEB1 ^3^ |
| ECM2 ^1^ | LAMA3 ^1^ | RGS4 ^1^ | CXCL8 ^1^ | PRSS8 ^3^ | ST14 ^3^ | MANSC1 ^3^ | C2orf15 ^3^ |  |
| EDIL3 ^1^ | LAMC1 ^1^ | RHOB ^1^ | P3H1 ^1^ | EXPH5 ^3^ | SSH3 ^3^ | ERBB2 ^3^ | SLC12A8 ^3^ |  |

**Table S2. Functional enrichment for differential expression associated with ex vivo culture (48 h control vs 0 h baseline).** Differential expression analysis was performed in edgeR using a paired design (patient as blocking factor) with TMM normalization (FDR*< 0.05, |log₂FC| ≥ 0.58).* Over-representation analysis was conducted in DAVID v2024 using a Homo sapiens background across GO (BP/CC/MF), KEGG, and Reactome. Columns report Count (genes per term), % (100 × Count/List Total as defined by DAVID), p-value, Benjamini–Hochberg adjusted q-value (BH-FDR), and fold enrichment (FE). Terms are listed exactly as exported by DAVID.

| **Upregulated** | | | | | | | |
| --- | --- | --- | --- | --- | --- | --- | --- |
| **Database** | **Term** | **Count** | **%** | **p-value** | **BH-FDR** | **FE** |  |
| GOTERM_BP_DIRECT | extracellular matrix organization | 6 | 31.6 | 0.000000 | 0.0001 | 37.5 |  |
| REACTOME_PATHWAY | **Extracellular matrix organization** | 7 | 36.8 | 0.000003 | 0.0005 | 14.7 |  |
| REACTOME_PATHWAY | **Degradation of the extracellular matrix** | 5 | 26.3 | 0.000035 | 0.0028 | 24.0 |  |
| GOTERM_BP_DIRECT | **positive regulation of smooth muscle cell proliferation** | 4 | 21.1 | 0.000016 | 0.0037 | 75.9 |  |
| REACTOME_PATHWAY | **Collagen degradation** | 4 | 21.1 | 0.000088 | 0.0047 | 42.3 |  |
| REACTOME_PATHWAY | **Integrin cell surface interactions** | 4 | 21.1 | 0.000204 | 0.0082 | 31.8 |  |
| GOTERM_CC_DIRECT | **focal adhesion** | 5 | 26.3 | 0.000450 | 0.0087 | 12.6 |  |
| GOTERM_BP_DIRECT | **blood coagulation** | 4 | 21.1 | 0.000111 | 0.0127 | 39.8 |  |
| REACTOME_PATHWAY | **Interleukin-4 and Interleukin-13 signaling** | 4 | 21.1 | 0.000460 | 0.0147 | 24.2 |  |
| REACTOME_PATHWAY | **Syndecan interactions** | 3 | 15.8 | 0.000625 | 0.0167 | 75.1 |  |
| KEGG_PATHWAY | Hematopoietic cell lineage | 4 | 21.1 | 0.000517 | 0.0335 | 22.8 |  |
| **Downregulated** | | | | | | | |
| **Database** | **Term** | **Count** | **%** | **P-Value** | **BH-FDR** | **FE** |  |
| GOTERM_CC_DIRECT | **extracellular matrix** | 5 | 38.0 | 0.00000 | 0.0001 | 42.4 |  |
| GOTERM_CC_DIRECT | **collagen-containing extracellular matrix** | 5 | 38.5 | 0.00002 | 0.0003 | 24.4 |  |
| GOTERM_MF_DIRECT | **extracellular matrix structural constituent** | 4 | 30.8 | 0.00003 | 0.0006 | 57.9 |  |
| REACTOME_PATHWAY | **Molecules associated with elastic fibres** | 3 | 23.1 | 0.00010 | 0.0026 | 155.3 |  |
| REACTOME_PATHWAY | **Elastic fibre formation** | 3 | 23.1 | 0.00014 | 0.0026 | 130.6 |  |
| GOTERM_CC_DIRECT | **extracellular region** | 7 | 53.9 | 0.00027 | 0.0027 | 5.7 |  |

**Table S3. Functional enrichment for differential expression after 15 µM selenite treatment (15 µM vs paired 48 h control).** Differential expression analysis was performed in edgeR using a paired design (patient as blocking factor) with TMM normalization (Benjamini–Hochberg (BH) FDR < 0.05; |log₂FC| ≥ 0.58). Over-representation analysis was conducted in DAVID v2024 using a Homo sapiens background across GO (BP/CC/MF), KEGG, and Reactome. Columns report Count (genes per term), % (100 × Count/List Total as defined by DAVID), p-value, Benjamini–Hochberg adjusted q-value (BH-FDR), and fold enrichment (FE). Terms are listed exactly as exported by DAVID.

| **Downregulated** | | | | | | |
| --- | --- | --- | --- | --- | --- | --- |
| **Database** | **Term** | **Count** | **%** | **p-value** | **BH-FDR** | **FE** |
| GOTERM_CC_DIRECT | **Basement membrane** | 8 | 61.5 | 3.0000E-14 | 7.0500E-13 | 131.9 |
| REACTOME_PATHWAY | **Extracellular matrix organization** | 8 | 61.5 | 1.4500E-09 | 1.1400E-07 | 25.9 |
| REACTOME_PATHWAY | **Laminin interactions** | 5 | 38.5 | 7.8300E-09 | 3.0900E-07 | 174.2 |
| GOTERM_CC_DIRECT | **Extracellular region** | 10 | 76.9 | 4.1300E-07 | 6.4600E-06 | 6.9 |
| REACTOME_PATHWAY | **ECM proteoglycans** | 5.0 | 38.5 | 3.6000E-07 | 9.4700E-06 | 68.7 |
| GOTERM_CC_DIRECT | endoplasmic reticulum lumen | 5 | 38.5 | 2.4100E-05 | 2.2700E-04 | 25.2 |
| KEGG_PATHWAY | **ECM-receptor interaction** | 4 | 30.8 | 3.7200E-05 | 3.1200E-04 | 47.9 |
| KEGG_PATHWAY | Small cell lung cancer | 4 | 30.8 | 4.2500E-05 | 3.1200E-04 | 45.9 |
| KEGG_PATHWAY | Amoebiasis | 4 | 30.8 | 5.7700E-05 | 3.1700E-04 | 41.4 |
| REACTOME_PATHWAY | **Non-integrin membrane-ECM interactions** | 4 | 30.8 | 3.9000E-05 | 7.7100E-04 | 51.6 |
| REACTOME_PATHWAY | Crosslinking of collagen fibrils | 3 | 23.1 | 1.0300E-04 | 1.6300E-03 | 174.2 |
| KEGG_PATHWAY | **Focal adhesion** | 4 | 30.8 | 4.3300E-04 | 1.9000E-03 | 21.0 |
| REACTOME_PATHWAY | Degradation of the extracellular matrix | 4 | 30.8 | 2.0400E-04 | 2.6800E-03 | 29.6 |
| KEGG_PATHWAY | Human papillomavirus infection | 4 | 30.8 | 1.8300E-03 | 6.7200E-03 | 12.8 |
| KEGG_PATHWAY | **PI3K-Akt signaling pathway** | 4 | 30.8 | 2.3300E-03 | 7.3300E-03 | 11.8 |
| REACTOME_PATHWAY | Assembly of collagen fibrils and other multimeric structures | 3 | 23.1 | 1.2100E-03 | 1.3700E-02 | 51.4 |
| KEGG_PATHWAY | Pathways in cancer | 4 | 30.8 | 7.0100E-03 | 1.9300E-02 | 8.0 |
| REACTOME_PATHWAY | Integrin cell surface interactions | 3 | 23.1 | 2.3400E-03 | 2.3000E-02 | 36.9 |
| REACTOME_PATHWAY | Collagen formation | 3 | 23.1 | 2.6200E-03 | 2.3000E-02 | 34.8 |
| REACTOME_PATHWAY | Post-translational protein phosphorylation | 3 | 23.1 | 3.8800E-03 | 3.0700E-02 | 28.5 |
| REACTOME_PATHWAY | Regulation of Insulin-like Growth Factor (IGF) transport and uptake by Insulin-like Growth Factor Binding Proteins (IGFBPs) | 3 | 23.1 | 5.1400E-03 | 3.6900E-02 | 24.7 |
